# NMR solution structure of tricyclo-DNA containing duplexes: insight into enhanced thermal stability and nuclease resistance

**DOI:** 10.1093/nar/gkz197

**Published:** 2019-03-27

**Authors:** Andrei Istrate, Silke Johannsen, Alena Istrate, Roland K O Sigel, Christian J Leumann

**Affiliations:** 1 Department of Chemistry and Biochemistry, University of Bern, Freiestrasse 3, Bern CH-3012, Switzerland; 2 Department of Chemistry, Winterthurerstrasse 190, University of Zürich, Zürich CH-8057, Switzerland

## Abstract

Tc-DNA is a conformationally constrained oligonucleotide analogue which shows significant increase in thermal stability when hybridized with RNA, DNA or tc-DNA. Remarkably, recent studies revealed that tc-DNA antisense oligonucleotides (AO) hold great promise for the treatment of Duchenne muscular dystrophy and spinal muscular atrophy. To date, no high-resolution structural data is available for fully modified tc-DNA duplexes and little is known about the origins of their enhanced thermal stability. Here, we report the structures of a fully modified tc-DNA oligonucleotide paired with either complementary RNA, DNA or tc-DNA. All three investigated duplexes maintain a right-handed helical structure with Watson-Crick base pairing and overall geometry intermediate between A- and B-type, but closer to A-type structures. All sugars of the tc-DNA and RNA residues adopt a North conformation whereas the DNA deoxyribose are found in a South-East-North conformation equilibrium. The conformation of the tc-DNA strand in the three determined structures is nearly identical and despite the different nature and local geometry of the complementary strand, the overall structures of the examined duplexes are very similar suggesting that the tc-DNA strand dominates the duplex structure.

## INTRODUCTION

Modified nucleic acids have shown widespread utility as diagnostic tools and oligonucleotide-based drugs. The key requirements for potential therapeutic oligonucleotides are resistance against nuclease degradation and high affinity to complementary nucleic acids. Therefore, great effort has been made to develop chemically modified oligonucleotides that are able to form Watson-Crick duplexes with increased thermal stability.

Many nucleic acid modifications are designed to modulate the conformation of the sugar-phosphate backbone, preorganizing them for duplex formation. This conformational preorganization often results in increased thermal stability and, as a consequence of the structural changes, to improved nuclease resistance. Prominent examples of such modifications are 2′-*O*-alkylated RNAs (1–3), hexitol nucleic acids (HNA) (4), 2′F-RNA (5), locked nucleic acids (LNA) (6,7) and the tricyclo-DNA family (tc-DNA) (8–12).

**Figure 1.**
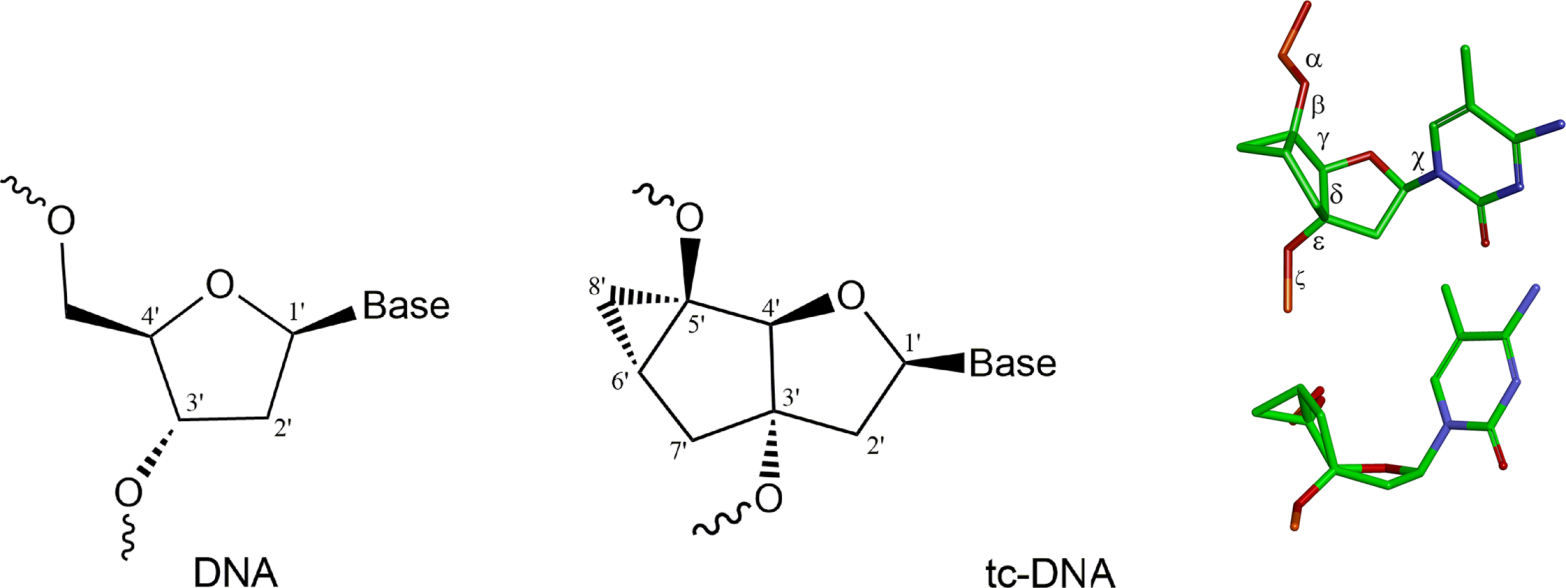
Chemical structures of DNA and tc-DNA. Atomic numbering and the dihedral angles along the phosphate-sugar backbone are indicated.

Tc-DNA is a conformationally constrained oligonucleotide analogue which deviates from natural DNA by three additional carbon atoms between C3′ and C5′ (Figure 1) (8). Fully modified tc-DNA oligomers show significantly increased thermal stability when hybridized with DNA (Δ*T*_m_/mod of 1.2°C), RNA (Δ*T*_m_/mod of 2.4°C) or tc-DNA (Δ*T*_m_/mod of 3.1°C) (13). Moreover, tc-DNA oligonucleotides are resistant to nuclease degradation and do not activate RNase H (14). Notably, recent studies revealed that tc-DNA antisense oligonucleotides (AO) hold great promise for the treatment of Duchenne muscular dystrophy (15) and spinal muscular atrophy (16) via an exon-skipping or inclusion mechanism.

Structural studies of modified oligonucleotides have provided valuable information on their conformational flexibility, thermal stability, and interaction with proteins (17–21). Very little is known about the structure of duplexes with tc-DNA modifications. CD spectroscopic studies and MD simulations showed that fully modified tc-DNA oligonucleotides form duplexes with DNA or RNA that adopt an overall A-like conformation, suggesting that tc-DNA is an RNA analogue (13). The only available high-resolution structure is the X-ray structure of a Dickerson–Drew DNA dodecamer containing single incorporations of tricyclo-adenosine in each strand. Although the duplex adopts an overall B-DNA conformation, the tc-A nucleotides feature a C2′*-exo* sugar pucker and adopt a glycosidic torsion angle of 164° consistent with an A-form conformation (22). Given the lack of comprehensive 3D-information, we decided to investigate the structural properties of nucleic acid duplexes containing fully-modified tc-DNA.

Here, we report for the first time on three high-resolution NMR solution structures of a fully modified tc-DNA oligonucleotide paired either with complementary RNA, DNA or tc-DNA. The presented structures were obtained by applying NOE-derived distance constraints during restrained MD (rMD) calculations. In addition, we have determined the sugar pucker preferences of all nucleotides by analysis of DQF-COSY spectra. To reveal the structural features of tc-DNA, we compare the calculated structures with each other and with canonical A-RNA and B-DNA duplexes. Finally, we review the origins of increased stability of duplexes containing tc-DNA and discuss the tc-DNA•RNA hybrid duplex with regard to RNase H activation.

## MATERIALS AND METHODS

### Synthesis and characterization of oligonucleotides

The fully modified tc-oligonucleotides 5′-tc(^Me^CT^Me^CGG^Me^CTTA^Me^C)-3′ and 5′-tc(GTAAG^Me^C^Me^CGAG)-3′ were synthesized by Synthena AG (Switzerland). The natural RNA oligonucleotide 5′-r(GUAAGCCGAG)-3′ was synthesized on the 10 μmol scale on a Pharmacia LKB Gene Assembler Special DNA Synthesizer using standard phosphoramidite solid-phase methods. The natural DNA oligonucleotides 5′-d(CTCGGCTTAC)-3′ and 5′-d(GTAAGCCGAG)-3′ were purchased from Microsynth AG (Switzerland). All oligonucleotides were purified by ion exchange HPLC using a DNAPac PA100 22 × 250 mm semipreparative column (Dionex) applying the following eluents: A (25 mM Tris, pH 7.0), B (25 mM Tris, 1.25 M NaCl, pH 7.0). The gradient was programed as follows: 15% of eluent B in A for 13 min, then 15% to 35% of eluent B in A in 11 min, then 100% B for 6 min The oligodeoxynucleotides were desalted using HiPrep 26/10 (GE Healthcare), characterized by ESI-MS, and lyophilized. Oligonucleotide concentrations were determined using a NanoDrop ND-100 UV−vis spectrophotometer (NanoDrop Technologies, Inc.).

### UV-melting studies

Thermal melting experiments were conducted at 260 nm on a Varian Cary 100-Bio UV−vis spectrophotometer (Varian Inc.) equipped with a Peltier element. The samples were prepared in 150 mM NaCl, 10 mM NaH_2_PO_4_ buffer at pH 7.0 with a duplex concentration of 2 μM. The temperature was varied in the range from 25 to 95°C for each duplex at the rate of 0.5°C/min. *T*_m_ values were obtained from the maximum of the first derivative curves using WinUV software. All *T*_m_ values are reported as the average of eight measurements.

### NMR spectroscopy

The tc-DNA•RNA, tc-DNA•DNA and tc-DNA•tc-DNA duplexes were prepared at 0.5 mM concentration in 50 mM NaCl and 10 mM NaH_2_PO_4_ buffer solution, pH 7.05. To prepare the samples, equimolar quantities of the complementary strands were combined and annealed by heating to 90°C and then slowly cooled to room temperature. NMR spectra were measured in either 99.990% D_2_O or 90% H_2_O/10% D_2_O on Bruker Avance 600 MHz spectrometer equipped with a 5 mm TCI CryoProbe™ and Bruker Avance 700 MHz equipped with a 5 mm TXI CryoProbe™. DQF-COSY, TOCSY (mixing time of 80 ms), ^13^C–^1^H HSQC, ^13^C–^1^H HMQC, ^1^H–^31^P HETCOR and NOESY (mixing time of 60, 100, 150 and 250 ms) spectra in D_2_O were recorded at 283 and 298 K. NOESY (mixing time of 250 ms) spectra in H_2_O were measured at 283 K in order to reduce exchange with water. All 2D spectra were processed by NMRPipe (23) and analyzed using SPARKY (24).

### NMR restraints

The cross peaks in NOESY spectra were integrated with the SPARKY program. The resulting volumes were divided into three classes based on peak overlap and intensity. Volumes derived from strong non-overlapping cross-peaks were included into the first class and were assigned to a 10% error level. Class two volumes were derived from slightly overlapped cross-peaks and were assigned to a 30% error level. Finally, low intensity and highly overlapped peaks were assigned to a 50% error level. Distance restraints were calculated from NOESY cross-peak volumes using complete relaxation matrix method employing a hybrid matrix approach with the MARDIGRAS (25) program. Calculations were performed using isotropic correlation times of 1, 2, 3 and 4 ns. Initial duplex models required for MARDIGRAS runs were built in A-form using the NAB (26) molecular manipulation language and subsequently energy minimized with the GROMACS 5.1 (27) software package. Since the MARDIGRAS results may depend on the starting structure, we repeated the distance restraints calculations using models obtained after several structure refinement iterations. The average width of the distance restraints was 0.92, 0.76 and 0.81 Å for tc-DNA•RNA, tc-DNA•DNA, and tc-DNA•tc-DNA duplexes respectively. The average restraint length was 4.40, 4.02 and 4.00 Å for tc-DNA•RNA, tc-DNA•DNA and tc-DNA•tc-DNA duplexes respectively.

Due to significant line widths (5–8 Hz) of the sugar proton signals in the DQF-COSY spectra, ^1^H–^1^H *J*-coupling constants could not be accurately measured. However, sugar puckering could be determined by analyzing the DQF-COSY cross-peak patterns and their fine structure.

### Restrained molecular dynamics calculations

Distance restraints calculated by MARDIGRAS, sugar puckering restraints as well as base pairing planarity and hydrogen bonding restraints were used during restraint molecular dynamics calculations (rMD) employing a simulated annealing protocol. The hydrogen bonding restraints were applied as distance restraint with the same weights as for the NOE restraints. Starting nucleic acid duplex structures in A- and B-form were created using the NAB language and energy minimized with the GROMACS 5.1 program using a steep descendent algorithm. The starting structures were subjected to *in vacuo* structure refinement with the CNS program using Cornell *et al.* force field (28) and the standard *anneal.inp* protocol of simulated annealing in Cartesian coordinates. The calculation protocol included two steps: slow cooling from 1000 to 0 K and final energy minimization of the structure using the Powell algorithm. Structures were visualized and analyzed with the use of the Pymol (29) program. The distance and dihedral angle restraints violations were monitored using the CNS program and in house written BASH and AWK scripts. The forcefield parameters for the modified nucleotides were adapted from existing parameters from the Cornell *et al.* force field and QM calculations at the HF/6-31G* theory level with the GAUSSIAN 09 (30) program. The R.E.D.-III.5 tools (31) program package was used to fit partial charges to each atomic centre according to the RESP (32) algorithm (see Supplementary Data).

An ensemble of 50 out of 100 structures with the lowest NOE violations resulting from *in vacuo* calculations were subjected to rMD in an explicit water environment with the GROMACS 5.1 program package and the Cornell *et al.* force field. Thus, the structures obtained after *in vacuo* refinement were placed in a cubic water box with ∼10 000 TIP3 model water molecules and 18 sodium ions to obtain an electroneutral system. The water molecules around the nucleic acid duplex were equilibrated by carrying out a 20 ps MD simulation with restrained positions of the nucleic acids atoms at 300 K. Next, the system was cooled from 300 to 0 K in 50 ps followed by two final energy minimizations with steep descendant and conjugated gradient algorithms. Ten structures were selected for the final structure family based on the NOE violation energy.

## RESULTS AND DISCUSSION

### Thermal stability

**Scheme 1.**
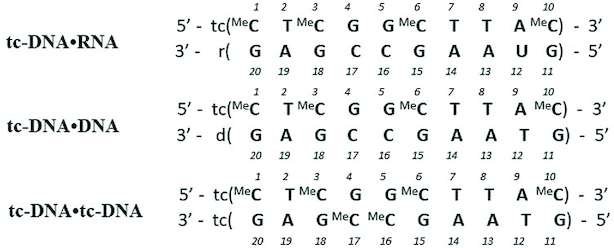
Base composition and numbering scheme of the three studied duplexes.

To examine the effect of tc-DNA on the conformation and properties of the modified duplexes, we prepared three duplexes in which a tc-DNA 10-mer oligomer was paired with either an RNA, DNA or tc-DNA complement. The sequences of the tc-DNA strand used in this study is a 10-nucleotide long part of a potent antisense oligonucleotide targeting the donor splice site of exon 23 of the mouse dystrophin pre-mRNA (15) (Scheme 1). In most cases, tricyclo oligonucleotides used in the antisense studies feature full C to ^Me^C substitutions to prevent the unwanted immune stimulation (33). In this context, we chose to use full C to ^Me^C substitutions in tc-DNA strands for our studies. First, we studied the thermal stability of the three studied duplexes by UV-melting experiments (Table 1). Compared to the natural DNA, tc-DNA oligonucleotide displayed increased affinities to both DNA and RNA complements (increase in *T*_m_ of +1.6°C and +2.7°C per tc-DNA nucleotide respectively). For the tc-DNA•tc-DNA duplex, an increase in *T*_m_ of 2.2°C per tc-DNA nucleotide was observed compared to the corresponding DNA•DNA and DNA•RNA duplexes. Overall, these results are consistent with the previously reported data on tc-DNA pairing properties (13).

**Table 1.** Melting temperatures and melting temperature change per modification (Δ*T*_m_/mod) of tc-DNA•RNA, tc-DNA•DNA, tc-DNA•DNA, DNA•DNA and DNA•RNA duplexes with identical base composition

Duplex	*T*_m_ (°C)	Δ*T*_m_/mod (°C)
DNA•DNA	45.0	
tc-DNA•DNA	61.3	+1.6
DNA•RNA	45.1	
tc-DNA•RNA	71.7	+2.7
tc-DNA•tc-DNA	88.1	+2.2

### Resonance assignment

**Figure 2.**
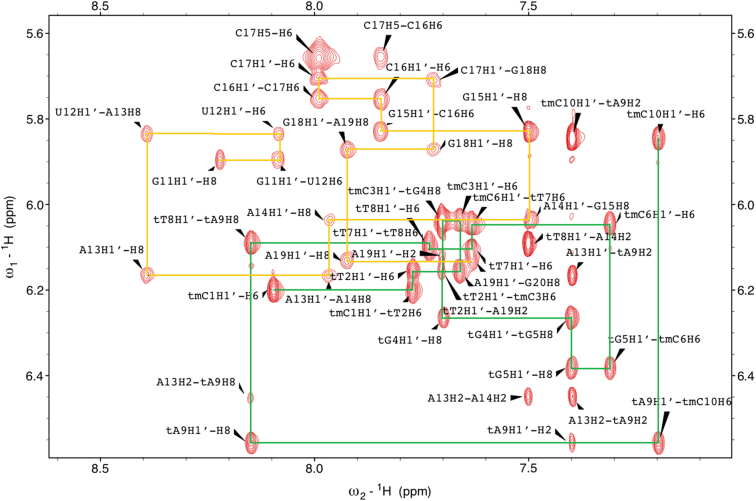
The aromatic to H1′ region of the 250 ms NOESY spectrum of tc-DNA•RNA hybrid. The sequential H8/6-H1′ connectivity pathways are indicated with green lines for the tc-DNA strand and yellow for the RNA strand.

The NOESY spectra of all three studied duplexes display cross-peaks characteristic for right-handed nucleic acid duplexes with Watson-Crick base pairing. The nonexchangeable protons were assigned using 2D NOESY, DQF-COSY, TOCSY, ^13^C-^1^H HSQC and ^13^C-^1^H HMQC spectra recorded at 298 K and following typical methods for double stranded nucleic acids (34–36). Sequential assignment of base H6/H8 protons and sugar H1′ protons was performed by the anomeric to aromatic proton walk in 2D NOESY spectra (Figure 2, Supplementary Figures S1 and S2). Adenosine H2 nucleobase signals were identified with the help of ^13^C–^1^H HMQC spectra based on different ^13^C chemical shifts for C6/C8 and C2 nucleobase carbons. Characteristic H6–H5 cross-peaks of cytidine and uridine as well as H6-Me7 of thymidine and 5-methylcytidine in DQF-COSY and TOCSY spectra were used to confirm the base identification. Determined base H6/H8 chemical shifts complemented by DQF-COSY, TOCSY and ^13^C–^1^H HSQC spectra were then employed in sugar signal assignment through H6/H8-H2′1, H2′2, H3′, H4′, H5′, H5″, H6′, H7′, H7″, H8′ and H8″ pathways in 2D NOESY spectra.

The exchangeable protons were assigned using the 2D NOESY spectra collected in H_2_O at 283 K. First, we identified the uridine and thymidine H3 imino protons by their characteristic strong correlation with the H2 protons of the adenosine base-pair complement. Next, the assignment of guanosine H1 imino protons was accomplished following the inter-strand H3(T2)-H1(G18)-H1(G4)-H1(G5)-H1(G15)-H3(T7)-H3(T8)-H3(T12 or U12) correlation pathways. Finally, the cross-peak analysis in the imino region resulted in assignment of the majority of the amino protons. Full assignments are given in Supplementary Data (Supplementary Tables S1–S8).

### Sugar conformation

To examine the conformations adopted by the nucleosides in the studied duplexes, we analyzed the ^3^*J*^1^_H–_^1^_H_-couplings of the sugar moieties. Due to the relatively large linewidth of the sugar proton signals (∼5–8 Hz) (Supplementary Figures S3–S5), the ^3^*J*^1^_H–_^1^_H_-couplings could not be accurately measured. However, the sugar conformations were determined using NOE peak intensities and rough estimates of the ^3^*J*-coupling constants from DQF-COSY spectra. The non-terminal tc-DNA nucleotides of the studied duplexes showed strong ^3^*J*_H1′-H2′2_ and undetectable 3*J*_H1′-H2′1_ couplings (Supplementary Figure S6A). This result indicates that the non-terminal tc-DNA nucleotides strongly prefer the North sugar pucker. This is consistent with the conformation adopted by tc-DNA units in the reported earlier X-ray structure of a DNA duplex with a single tc-DNA incorporation (22). The terminal tc-DNA nucleotides exhibited strong ^3^*J*_H1′–H2′2_ and weak ^3^*J*_H1′–H2′1_ couplings, which indicates an equilibrium between Northern and Southern conformers. The population of the Northern conformer was estimated to 70–80% based on analysis of the peak fine structure. We next analyzed the sugar puckers of the RNA strand within the tc-DNA•RNA duplex. The DQF-COSY spectrum revealed strong ^3^*J*_H3′–H4′_ and undetectable 3*J*_H1′–H2′_ couplings for all nonterminal residues which indicates that the RNA nucleosides strongly favor the Northern conformation (Supplementary Figure S6B).

The deoxyribonucleotides in the tc-DNA•DNA duplex showed a combination of strong ^3^*J*_H1′–H2′1_, medium ^3^*J*_H1′–H2′2_ and ^3^*J*_H2′1–H3′_, and very weak or undetectable 3*J*_H2′2–H3′_ couplings, which point to the preference for the South conformation of the DNA sugars (Supplementary Figure S7A and B). However, we observed medium ^3^*J*_H3′–H4′_ instead of very weak ^3^*J*_H3′–H4′_ coupling constants (for eight out of ten residues), expected for a full South conformation (Supplementary Figure S7C). This suggests the existence of either a South-North, South-East or South-East-North equilibrium in which the South conformation is the most populated form. By comparison with simulated DQF-COSY spectra (37), we estimated the rate of the South conformation to be 70–80% for non-terminal residues and approximatively 60% for terminal residues. The H1′–H4′ NOE distances could be used to discriminate between the three possible equilibriums, as a South (70–80%)/North conformer mixture would yield distances of 3.0–3.3 Å, whereas a South(70–80%)/East mixture would result in distances of 2.6–2.8 Å. However, the analysis of the NOE intensities revealed that the H1′-H4′ NOE distances are intermediate (*d*_H1′–H4′_ = 2.9–3.1 Å), which supports the existence of a South-East-North deoxyribose conformation equilibrium. The high conformational flexibility of the DNA strand is not unexpected, and was observed previously in NMR studies of hybrid duplexes of DNA with oligonucleotides which prefer North sugar conformation, such as RNA or LNA (38–42).

### Structure calculation

The 3D structures were calculated employing the distance restraints obtained from 2D NOESY NMR spectra and the dihedral angle restraints derived from the sugar conformation analysis (Table 2, Supplementary Figures S8–S10), as described in the ‘Material and methods’ section. Dihedral angle restraints were defined only for the sugars of the non-terminal residues. To the non-terminal tc-DNA and RNA furanose rings were assigned dihedral angle restraints with values characteristic for the Northern part of the pseudorotation cycle (−25° ±10° and −35° ±10° for *ν*_1_ and *ν*_3_, respectively). Loose dihedral angle restraints typical for the South conformation were defined for the non-terminal DNA residues except dC16. Only *ν*_1_ dihedral angle of the dC16 residue was restraint because the H3′ proton chemical shift could not be assigned. In addition, considering that ^3^*J*_H4′-H5′1_ and ^3^*J*_H4′–H5′2_ of the DNA and RNA strands were very weak or undetectable, the γ angles of non-terminal DNA and RNA residues were restrained to the *gauche+* range (60° ± 30°). Since the ^1^H–^31^P HETCOR spectra showed low peak dispersion and thus the coupling constants could not be estimated, other backbone dihedral angles were not restrained (Supplementary Figure S11). In addition to the experimental restraints, eight planarity restraints were defined for nonterminal residues in order to keep the base pairs planar during the calculations. The weight of planarity restraints was 25 kcal mole^−1^ Å^−1^. Imino regions of the 2D NOESY spectra exhibit signals typical for nucleic acids duplexes with Watson–Crick base pairing. Eight imino signals corresponding to the nonterminal base pairs were assigned for the tc-DNA•RNA, and tc-DNA•tc–DNA duplexes and nine for the tc-DNA•DNA duplex. In addition to the non-terminal base pairs imino protons, the dG11-H1 terminal proton was assigned (Supplementary Figures S12–S17). To maintain Watson-Crick base pairing during structure calculations we applied 20 distance restraints corresponding to the hydrogen bonds for the non-terminal residues of the tc-DNA•RNA, and tc-DNA•tc-DNA duplexes and 23 distance restraints (non-terminal residues and tcC10 and dG11 terminal residues) for the tc-DNA•DNA duplex.

**Table 2.** Number of restraints used for the structure calculation and the statistics of the calculated families

Parameter	tc-DNA•RNA	tc-DNA•DNA	tc-DNA• tc-DNA
NOE restraints	743	621	377
Intra-residual NOE	376	370	204
Inter-residual NOE	367	251	173
Hydrogen bonding restraints	20	23	20
Sugar pseudorotation restraints	36	36	36
Base pair planarity restraints	16	16	16
Total number of restraints	816	696	449
Number of conformers in a family of structures	10	10	10
Number of NOE violations (>0.5 Å) per structure	0	0	0
Number of torsion angle (>5°) per structure	0	2	0
Pairwise all heavy atom RMSD (Å)			
Both strands	0.54	0.46	0.48
Strand1	0.38	0.27	0.30
Strand2	0.47	0.32	0.33

**Figure 3.**
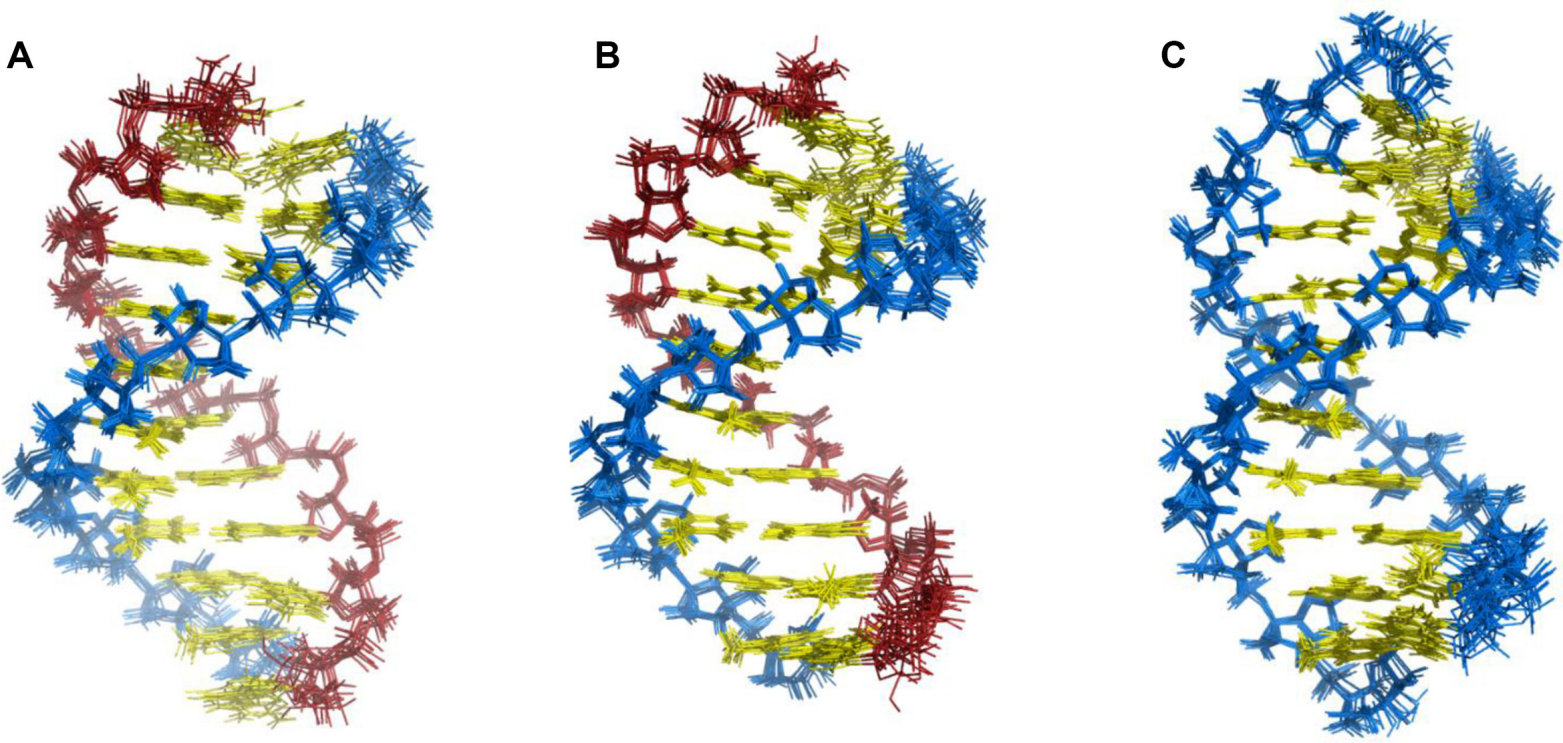
Comparison of the (**A**) tc-DNA•RNA, (**B**) tc-DNA•DNA and (**C**) tc- DNA•tc-DNA ensembles of the 10 lowest energy structures each. The nucleobases are shown in yellow, tc-DNA sugar-phosphate backbones are shown in blue, and DNA/RNA sugar-phosphate backbones are shown in red.

The resulting structural families of the tc-DNA•RNA, tc-DNA•DNA and tc-DNA•tc-DNA duplexes are shown in Figure 3 and Supplementary Figure S18. All three structural families exhibit good convergence with pairwise RMSD between 0.46 and 0.54 Å (excluding the terminal residues). The obtained structure ensembles feature low restraint violations. Specifically, no NOE violations >0.5 Å were observed in any of the final structures (Table 2).

### Structure description

The tc-DNA•RNA, tc-DNA•DNA and tc-DNA•tc-DNA duplexes maintain a right-handed helical structure with Watson-Crick base pairing (Figure 3). The three obtained structural families exhibit high similarity to each other, with an average heavy atom RMSD of 1.2 Å (excluding the terminal residues) between the representative structures. To examine the variability of the tc-DNA strand among the studied duplexes, we calculated the average heavy atom RMSD of the tc-DNA strand from all the duplexes. Since tc-DNA, RNA and DNA chains have different number atoms, only heavy atoms present in both compared strands were used for pairwise RMSD calculations. Interestingly, the obtained RMSD value was 0.7 Å, which is close to the RMSD values within the distinct structural families (0.46–0.54 Å). Hence, the geometry of the tc-DNA strands in tc-DNA•RNA, tc-DNA•DNA and tc-DNA•tc–DNA duplexes is very similar. In contrast, the RNA, DNA, and tc-DNA complementary strands exhibit local differences when compared with each other, which leads to higher RMSD values (1.22–1.73 Å, Table 3). Although the overall structure of the tc-DNA•RNA, tc-DNA•RNA and tc-DNA•tc-DNA duplexes is neither a canonical A- nor B-type, it resembles an A-form helix. Indeed, the RMSD between the representative structures of the calculated structural families and the canonical A-form duplex ranges from 1.76 to 1.96 Å. In contrast, when compared to the B-form duplex, the RMSD has considerably higher values (2.51–2.86 Å). As expected, out of the three duplexes, the tc-DNA•RNA duplex exhibits the lowest RMSD when superimposed with the canonical A-form helix 1.76 Å (Table 3).

**Table 3.** RMSD statistics derived from superposition of the representative structures (excluding the four terminal residues) of the calculated ensembles with each other and with canonical B-DNA and A-RNA duplexes

	Pairwise RMSD of heavy atoms (Å)		
Compared structures	Strand 1	Strand 2	Both strands
tc-DNA•RNA and tc-DNA•DNA	0.70	1.35	1.12
tc-DNA•RNA and tc-DNA•tc-DNA	0.84	1.73	1.48
tc-DNA•tc-DNA and tc-DNA•DNA	0.60	1.22	1.03
tc-DNA•RNA and A-RNA	1.47	1.3	1.76
tc-DNA•DNA and A-RNA	1.43	1.54	1.87
tc-DNA•tc-DNA and A-RNA	1.47	1.54	1.96
tc-DNA•RNA and B-DNA	2.06	2.08	2.86
tc-DNA•DNA and B-DNA	1.86	1.54	2.51
tc-DNA•tc-DNA and B-DNA	1.82	1.86	2.53

**Figure 4.**
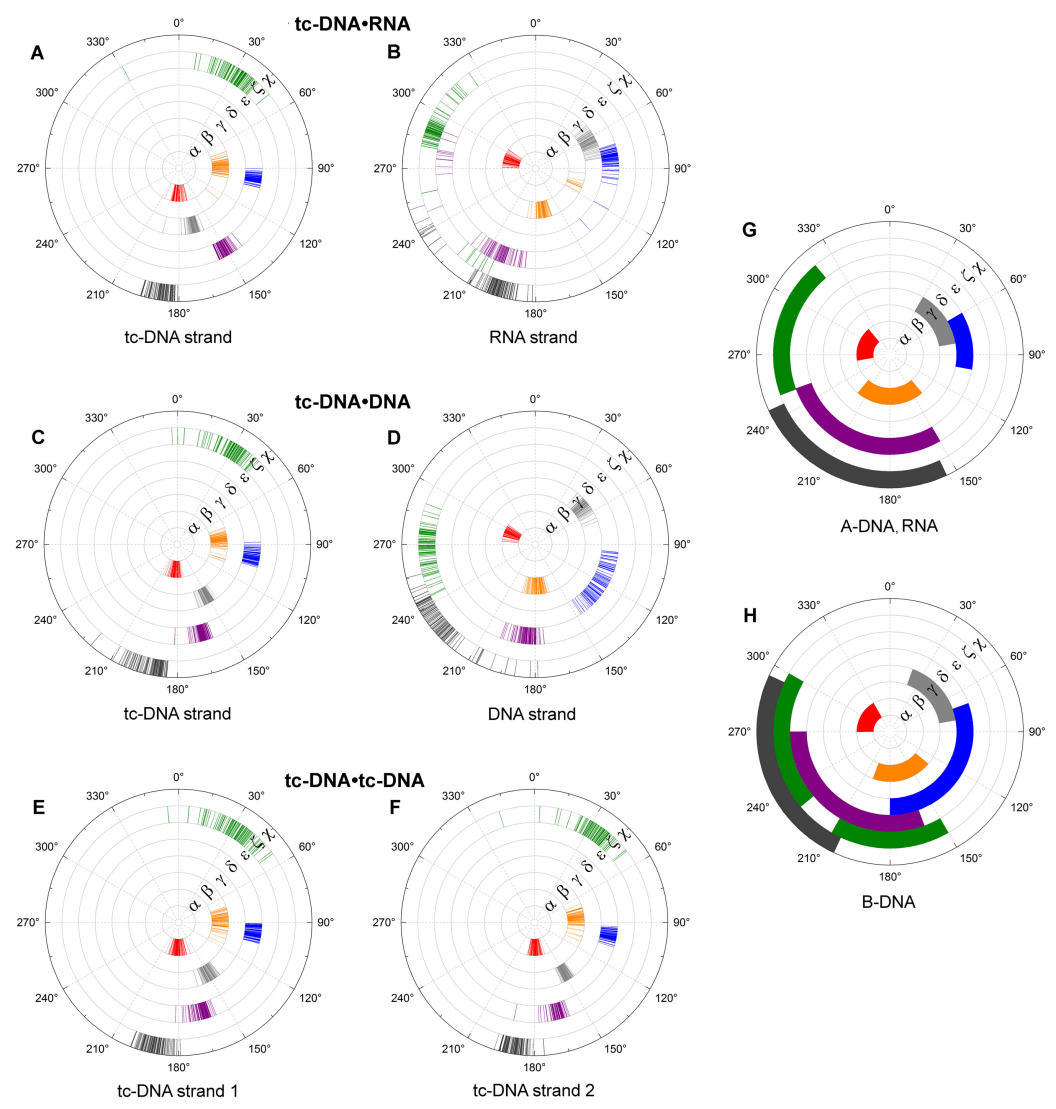
Backbone thorsion angle and glycosidic angles of the final tc-DNA•RNA (**A, B**) tc-DNA•DNA (**C, D**) and tc-DNA•tc-DNA (**E, F**) compared to the corresponding values found in A- and B-DNA structures (**G, H**).

Figure 4 illustrates the backbone torsion angle distribution for the studied duplexes as well as the data for the canonical A- and B-type helices (43). Consistent with previous observations, the tc-DNA backbone torsion angles (α, β, γ, δ, ϵ and ζ) adopt very similar values within all three duplexes (Figure 4A, C, E and F). Specifically, the phosphodiester bonds (torsion angles α and ζ) fall into the *-ap/+sc* range in contrast to *-sc/-sc* arrangement typically preferred by A- and B-form duplexes. Moreover, the fused ring system of the tc-sugars greatly affects the β, γ, and ϵ torsion angles, while retaining the δ angle in the ac^+^ range typical for A- or B-type structures. More specifically, the β, γ, and ϵ angles of the tc-strands fall into the sc^+^, ap^+^, and ap^+^ ranges, respectively, in contrast to the values found in the A- (ap^±^, sc^+^, and ac^−^ for β, γ, and ϵ) and B-helices (ap^±^, sc^+^, and ac^−^ to ap^±^ for β, γ, and ϵ). The values for the backbone torsion angles of the RNA strand fall into the same conformational ranges as in A-RNA (sc^−^, ap^+^, sc^+^, sc^+^, ap^−^ and sc^−^ for the angles α, β, γ, δ, ϵ and ζ) (Figure 4B). In a similar fashion, the backbone torsion angles of the DNA strand adopt values typical for a B-type conformation (sc^−^, ap^±^, sc^+^, ac^+^, ap^±^ and sc^−^ to ac^−^ for the angles α, β, γ, δ, ϵ and ζ) (Figure 4D). The glycosidic torsion angles of tc-DNA and RNA residues range from −170° to −160°, and correspond to the χ values of A-form oligonucleotide duplexes and RNA•DNA hybrid duplexes (40,43). Furthermore, the glycosidic torsion angles of the DNA strand range from −115° to −135°, which is typical for DNA in B-type duplexes and in RNA•DNA hybrid duplexes.

We next analysed the helical parameters of the modified duplexes with respect to the A- and B-type structures (Table 4). The *x*-displacement of the modified duplexes adopts average values between −2.8 and −3.4 Å, which is close to values found in A-type helices (−4.2 to −5.2 Å). The average helical rise is 3.1 Å, which is intermediate compared with the values found in A-RNA (2.8 Å) and B-DNA (3.3 Å). The helical twist is largely dependent on the complementary strand. Thus, when the tc-DNA strand is paired with RNA, the helical twist adopts values typical for an A-form helix, whereas the helical twist of tc-DNA•DNA and tc-DNA•tc-DNA duplexes adopts values intermediate between those observed in A- and B-form structures.

**Table 4.** Average helical parameters for calculated structure ensembles. Values for RNA•DNA and canonical A- and B-type duplexes are included for comparison

Parameter	tc-DNA•RNA	tc-DNA•DNA	tc-DNA•tc-DNA	RNA•DNA (40,44)	A-RNA	B-DNA
x displacement (Å)	−3.4 ± 0.8	−2.8 ± 0.7	−2.9	−4.0	−5.2	0.1
Rise (Å)	3.05 ± 0.3	3.1 ± 0.3	3.1	2.9	2.8	3.3
Twist (°)	32 ± 1.9	34.2 ± 2.6	35.6	31.3	32.7	36.5
Minor groove (Å)	7.3 ± 0.5	6.0 ± 0.5	5.4	9.5	10.9	5.7
Major groove (Å)	13.1 ± 0.8	13.5 ± 0.7	13	8.4	3.8	11.7
P-P distance (Å)	7.1 ± 0.1 tc-DNA	7.1 ± 0.1 tc-DNA	7.1	5.9 DNA	5.9	7
	6.2 ± 0.1 RNA	6.5 ± 0.3 DNA		5.4–6.6 RNA		

**Figure 5.**
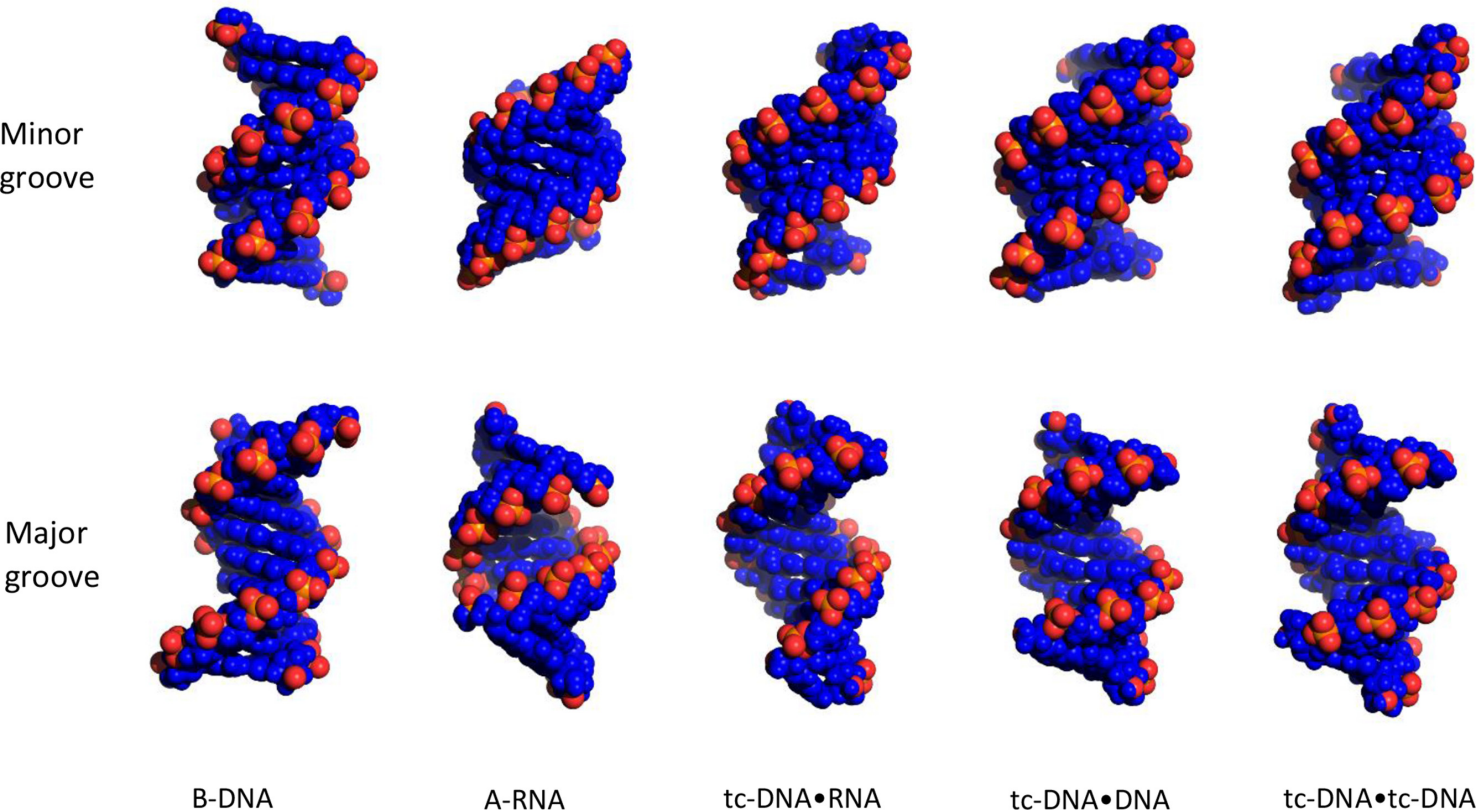
Minor and major grooves of the determined structures in comparison with canonical A- and B-type duplexes.

The structural features of the minor and major grooves of the nucleic acid duplexes are particularly important for the understanding of nucleic acid-protein interactions. In the studied tc-DNA•RNA, tc-DNA•DNA, and tc-DNA•tc-DNA duplexes, the major groove is wider than in both, A- and B-type structures. However, it is very deep which is consistent with the A-type conformation (Figure 5 and Table 4). The minor groove depth is intermediate between those of canonical A- and B-type conformations. In A- and B-type duplexes, the phosphate groups are oriented towards the major groove (45,46). In contrast to that, the phosphate groups of the tc-DNA strand are oriented towards the minor groove which can be explained by the restrained conformation of the tc-sugar. As a result, the size of the minor groove in the determined structures largely depends on the type of the chain complementary to tc-DNA, and varies from 5.4 Å (tc-DNA•tc-DNA) to 6.0 Å (tc-DNA•DNA) and 7.3 Å (tc-DNA•RNA) (Table 4).

### RNase H resistance of tc-DNA•RNA duplex

The determined structure of the tc-DNA•RNA duplex explains the inability of the fully-modified tc-DNA•RNA duplexes to elicit RNase H activity (14). Previously, several studies provided valuable insights into how RNase H discriminates DNA•RNA hybrid duplexes from DNA and RNA duplexes. Fedoroff *et al.* (39) hypothesized that RNase H recognizes the minor groove of duplexes with widths that are intermediate between those observed for A- and B-type duplexes (8–9 Å). Indeed, further X-ray crystallography studies revealed that the active site of RNase H has two grooves separated by 8.5 Å, which accommodate the backbones of a DNA•RNA hybrid. Moreover, upon binding with RNase H, the DNA phosphate group located two base-pairs away from the scissile bond is placed in a spatially conserved pocket. This leads to a large distortion of the α- and γ-backbone torsion angles (by ∼150°) (47,48). Moreover, to ensure the efficient cleavage of the RNA strand, the DNA sugars of the antisense strand are forced to adopt different conformations (C3′*-endo*, C2′*-endo* or O4′*-endo*) depending on their position in the binding site of RNase H (49). Therefore, the DNA strand flexibility is crucial for RNase H cleavage. The analysis of the determined structures shows that the minor grove in the tcDNA•RNA duplex (7.3 Å) is significantly narrower than the distance typically recognized by RNase H (8.5 Å). Moreover, the tc-DNA backbone is very rigid because of the restraint conformation of the tc-sugar. These structural features of the tc-DNA•RNA hybrid lead to its resistance to RNase H degradation.

### The origins of increased stability of duplexes containing tc-DNA

High affinity and selectivity of AOs towards complementary RNA are essential for their practical application. In this context, it is important to understand the origins of the enhanced thermal stability of the herein studied tc-DNA modified duplexes. There are a number of other prominent examples of NA modifications that are known to increase NA thermal stability, including LNA, 2′-F-RNA and HNA. In this respect, it is interesting to compare the structural properties of the tc-DNA modified duplexes with LNA, 2′-F-RNA and HNA.

**Figure 6.**
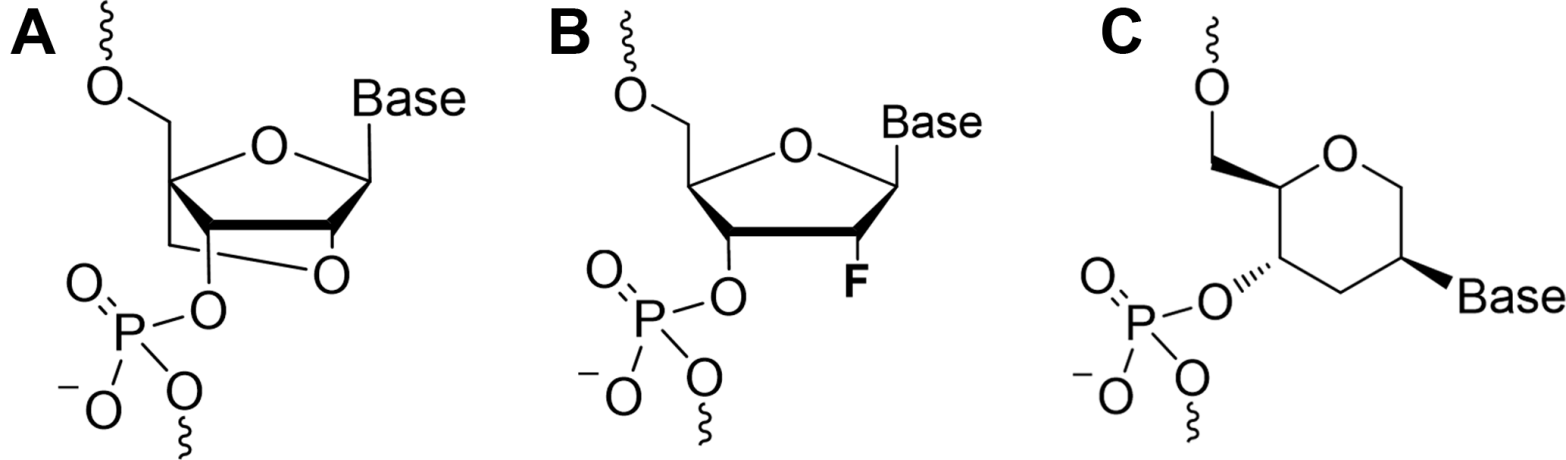
Chemical structure of (**A**) Locked nucleic acids (LNA), (**B**) 2′-F-RNA and (**C**) Hexitol nucleic acids (HNA).

LNA is a nucleic acid analogue which contains a 2′-O,4′-C methylene linkage (Figure 6A). The methylene linkage locks the LNA sugar in an N-type (C3′-endo) conformation. The fully modified LNA•RNA hybrid adopts an A-like conformation which is intermediate between RNA•RNA and LNA•LNA. The methylene bridges of the LNA nucleotides are located at the edge of the minor groove where they do not impose steric hindrance for duplex formation. The enhanced thermal stability of the LNA•RNA duplexes is attributed to reduced entropy loss upon binding due to the preorganization of the sugar conformation. Another stabilization factor is the improved minor grove hydration as a result of water-bridge formation between the O2′ of one residue and O4′ of the 3′-flanking sugar (21,50).

Another well-studied RNA analogue is 2′-F-RNA (Figure 6B). In a recent work, Egli *et al.* have extensively studied the structure of 2′-F-RNA modified oligonucleotide duplexes (19). By comparing the structure of partially or fully 2′-F-modified RNA duplexes with the reference RNA duplex they demonstrated that substitution of the 2′-OH group with fluorine is of little consequence in terms of the local and overall helix geometry. Unlike in the case of LNA-modified duplexes, the increased thermal stability of 2′-F-RNA modified duplexes was shown to be almost exclusively due to a favorable enthalpy change. The authors proposed that the presence of the strongly electronegative fluorine leads to increased strength of W–C hydrogen bonds and enhanced base staking which arises from long-range effects of the fluorine on the neighboring nucleobase. In a later work, Gonzalez and co-workers demonstrated that fluorine-enhanced FC-H···O backbone interactions are an additional stabilizing factor (51).

HNA are modified nucleic acids that contain an extra methylene group inserted between C1′ and O4′ of the underlying sugar, compared to DNA (Figure 6C). Considerable efforts have been made to investigate the structure of HNA nucleic acids (52–54). In particular both, solution NMR- and X-ray structures are available for HNA•RNA duplexes (52,54). The data show that HNA•RNA duplexes adopt A-like conformations both in solution and under high salt concentration in the crystalline state. However, there are some differences: the average helical twist is 3–5° lower in the X-ray structure, and the x-displacement is −5 Å in the X-ray structure compared to −3.4 Å in the NMR solution structure. The crystallographic asymmetric unit contains four distinct HNA•RNA hybrid duplexes. Although they only slightly differ in their overall helical conformation, there are significant local structural perturbations which point to a considerable structural flexibility of HNA•RNA duplexes. The authors revealed two main factors which contributes to increased thermal stability of the HNA•RNA hybrid duplex compared to RNA•RNA duplex. The first factor is structure preorganization as a result of the conformational constraints imposed by the six-membered ring. The second factor is reinforced hydration due to tighter bridging of the adjacent O2P phosphate atoms by water molecules in the HNA strand.

The above mentioned modifications result in enhanced thermal stability of the NA duplexes compared to natural DNA and RNA duplexes. In the case of LNA and HNA, the main stabilizing factors are structure preorganization and improved hydration. In contrast, 2′-F-RNA stabilizes the duplex structure by increasing strength of W-C hydrogen bonds, enhanced base staking, and enhanced of FC-H···O backbone interaction.

**Figure 7.**
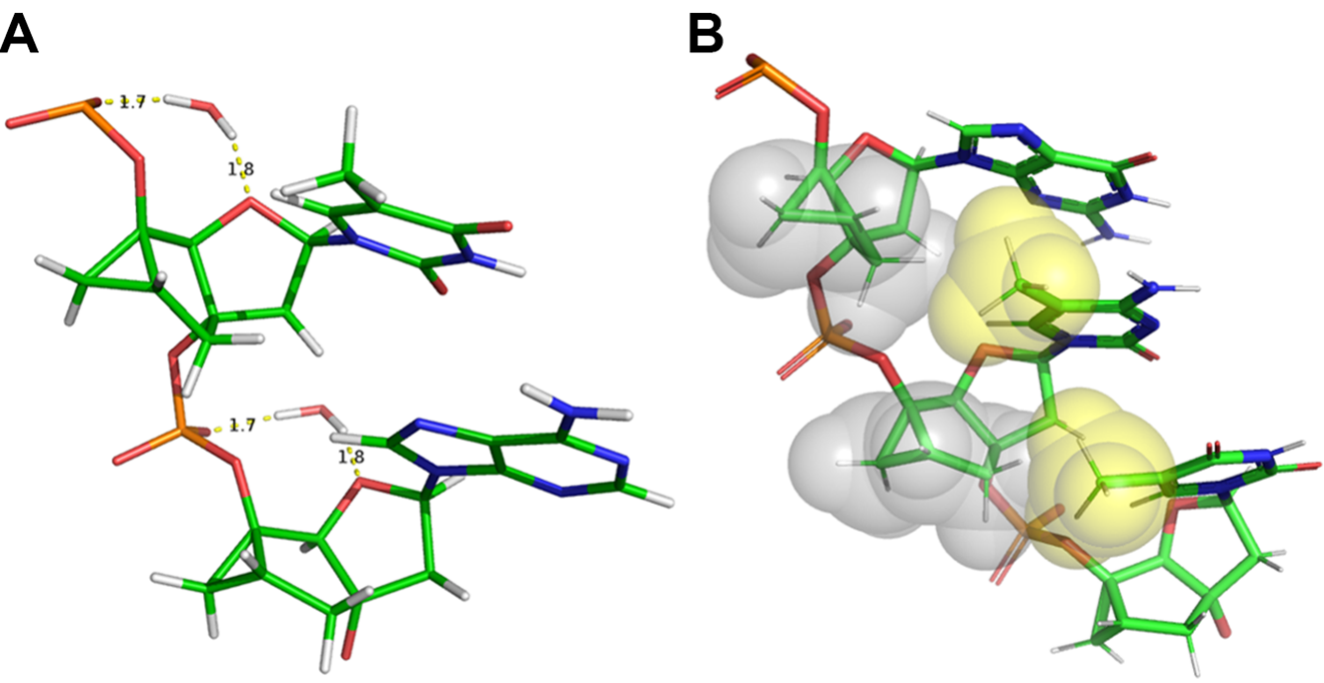
(**A**) View of water bridges between O1P phosphate and O4′ sugar atoms of tc-DNA strand in the tc-DNA•RNA duplex. (**B**) Space-filling, fragment of tc-DNA•RNA duplex showing close hydrophobic contacts between nucleobases methyl groups (yellow) and tc-DNA sugars (gray).

Similarly to LNA and HNA, the sugar of the herein examined tc-DNA modification is restrained to the Northern conformation. Moreover, the γ-backbone angle of tc-DNA is also constrained. As a result, the tc-DNA oligonucleotide is significantly less flexible than natural oligonucleotides, which leads to reduced entropy loss upon duplex formation. Interestingly, the tc-DNA•RNA and tc-DNA•DNA hybrid duplexes are somewhat intermediate between fully modified tc-DNA•tc-DNA and unmodified DNA•DNA and DNA•DNA duplexes. This is similar to the structure properties of the LNA•RNA hybrid which is intermediate between LNA•LNA RNA•RNA (50). As was mentioned above, hydration plays an important role in NA duplex stability. NMR spectroscopy does not provide direct information on NA duplexes hydration. However, a close examination of the determined structures reveals that tc-DNA strands would allow for intranucleotide water-bridge formation between O1P phosphate and O4′ sugar atoms, which may be an additional stabilization factor (Figure 7A). In addition, the hydrophobic parts of the tc-DNA sugars are in close contact with the nucleobases. This leads to the formation of a hydrophobic core, which may entropically favor the duplex stability (Figure 7B).

## CONCLUSIONS

In this work, we have studied three decamer duplexes each containing a fully modified tc-DNA strand, namely a tc-DNA•RNA duplex, a tc-DNA•DNA duplex, and a tc-DNA•tc-DNA duplex. The structures of the investigated oligonucleotide duplexes can be described as intermediate conformations between canonical A- and B-type, but are closer to A-type structures. All non-terminal tc-DNA residues show a strong preference for the North sugar pucker. Moreover, the conformations of the tc-DNA strands in all three structures are nearly identical, which is most likely a consequence of the high rigidity of the tc-sugars and the constrained γ-backbone torsion angle. Despite the differences in the backbone torsion angles, the glycosidic angle and the sugar puckering of the complementary strands, the overall helical conformation of the three duplexes is very similar, suggesting that the tc-DNA strand dominates the duplex structure. A detailed analysis revealed the existence of water bridges between O1P and O4′ of tc-DNA sugar atoms, which together with increased hydrophobic interactions and the structural preorganization of the tc-DNA strands may explain the enhanced stability of the tc-DNA duplexes.

## DATA AVAILABILITY

Atomic coordinates have been deposited in the Protein Data Bank with the accession codes 6GPI, 6GN4 and 6GMY.

## Supplementary Material

Supplementary Data
