# Supplementary material for: NMR solution structure of tricyclo-DNA containing duplexes: insight into enhanced thermal stability and nuclease resistance
Source: Nucleic Acids Res. 2019 Mar 27;47(9):4872–82. doi: 10.1093/nar/gkz197 (PMC6511864; doi:10.1093/nar/gkz197)
Supplement: Supplementary Data [file gkz197_supplemental_files.pdf]

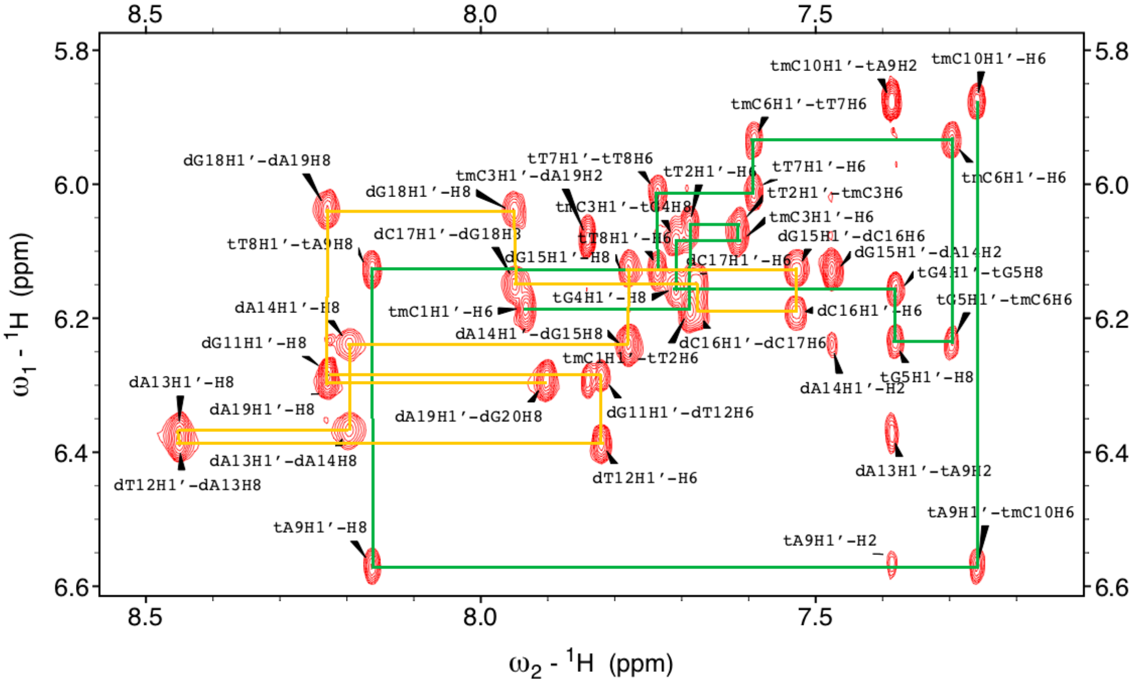

Figure S1. The aromatic to H1' region of the 250 ms NOESY spectrum of tc-DNA•DNA hybrid. The sequential H8/6-H1' connectivity pathways are indicated with green lines for the tc-DNA strand and yellow for the DNA strand.

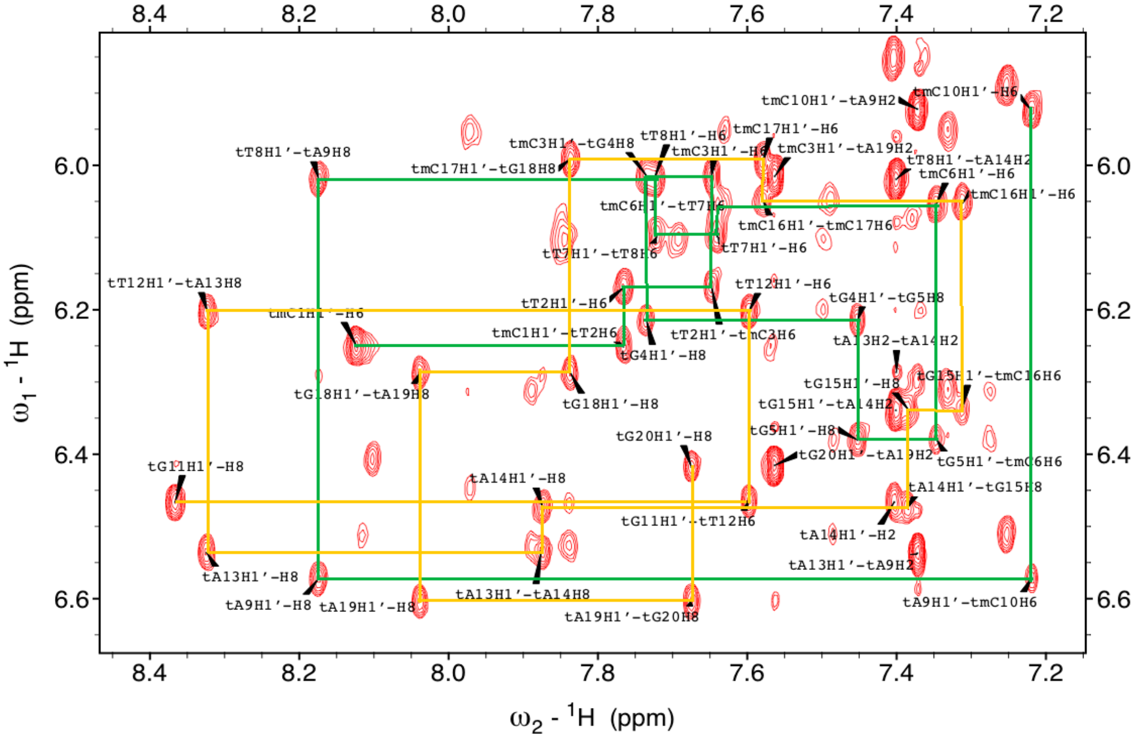

Figure S2. The aromatic to H1' region of the 250 ms NOESY spectrum of tc-DNA•tc-DNA duplex. The sequential H8/6-H1' connectivity pathways are indicated with green lines for the first tc-DNA strand and yellow for the second tc-DNA strand.

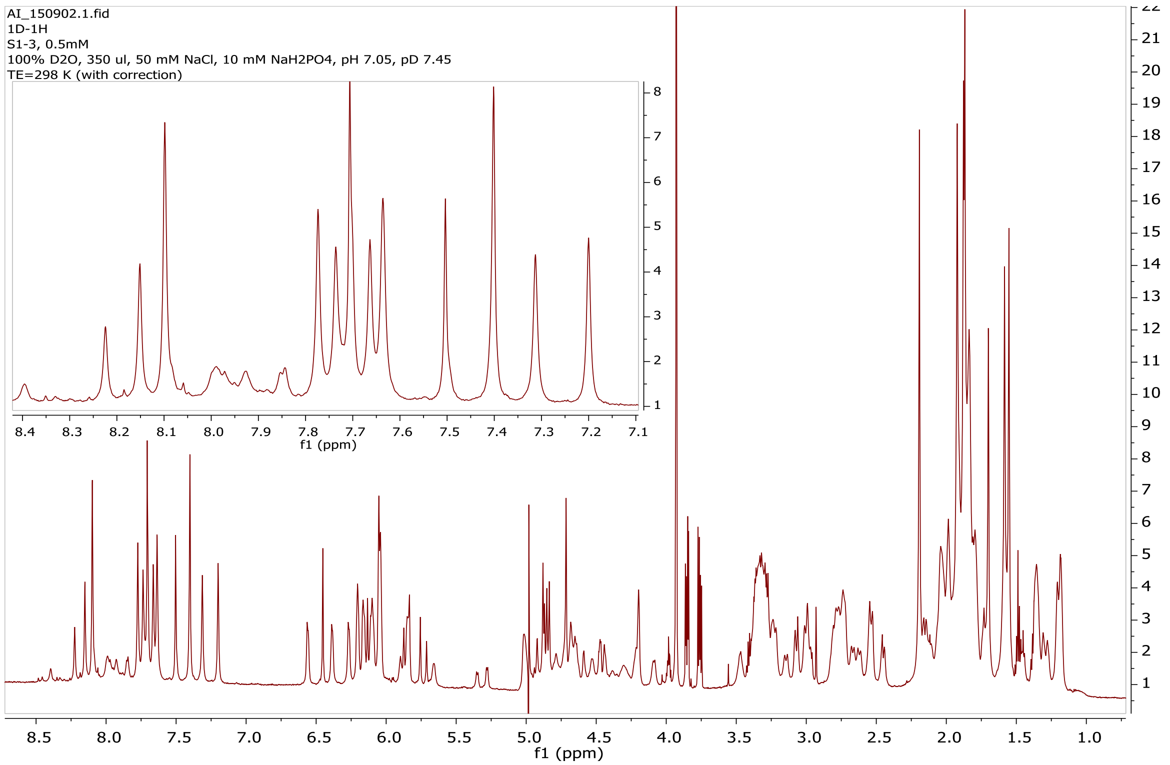

Figure S3. One-dimensional NMR spectrum of tc-DNA•RNA duplex in D2O at 298 K.

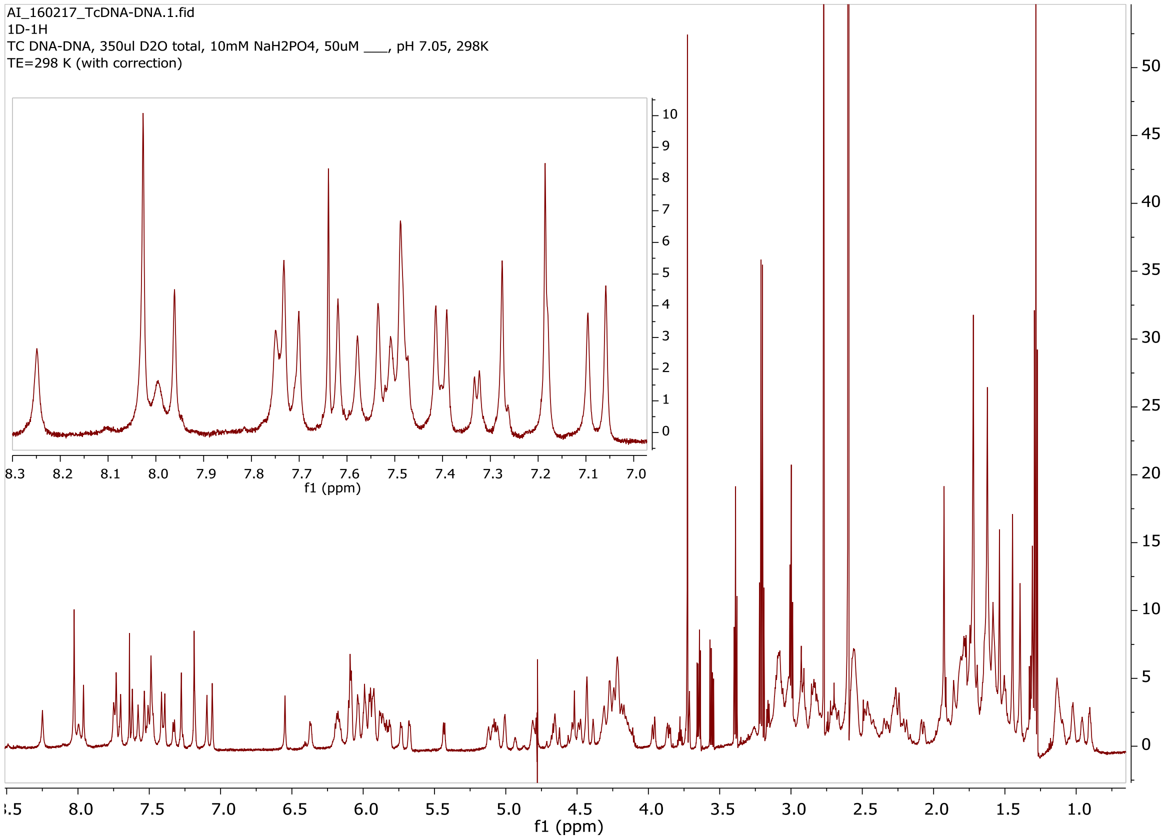

Figure S4. One-dimensional NMR spectrum of tc-DNA•DNA duplex in D2O at 298 K.

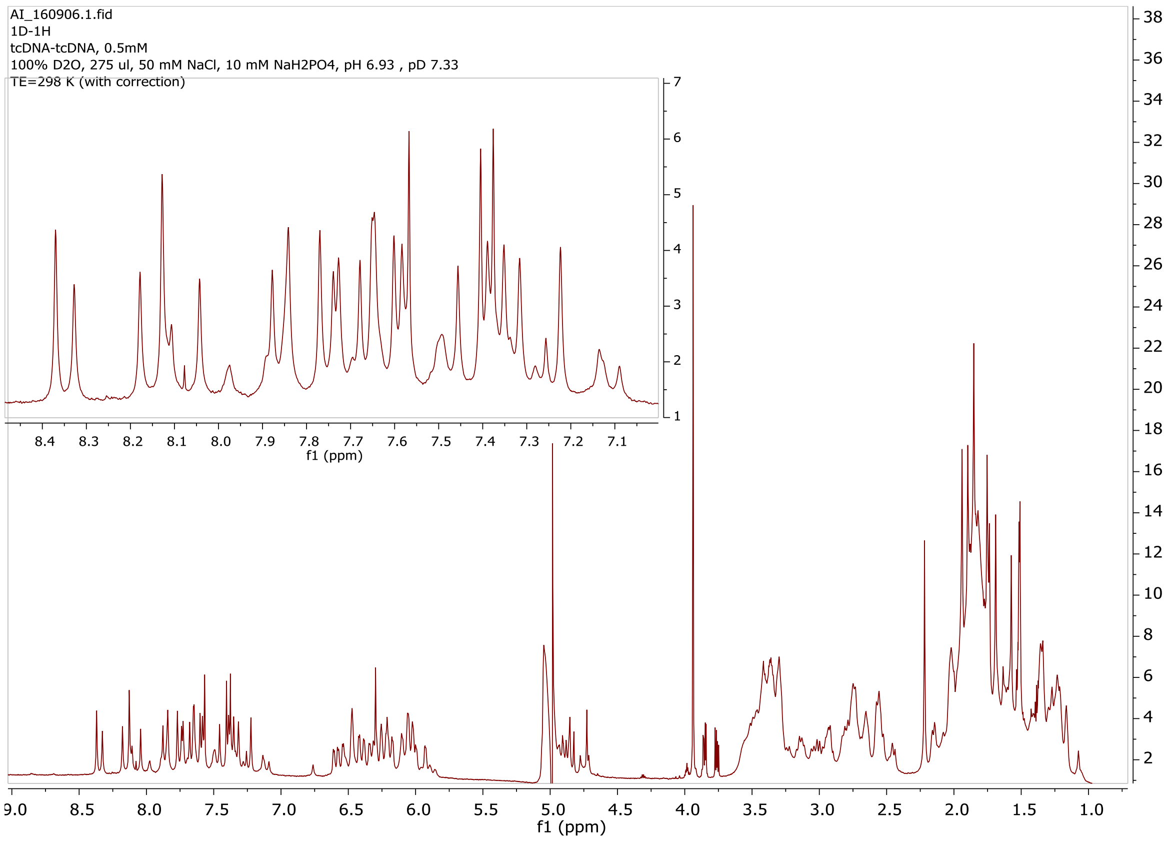

Figure S5. One-dimensional NMR spectrum of tc-DNA• tc-DNA duplex in D2O at 298 K.

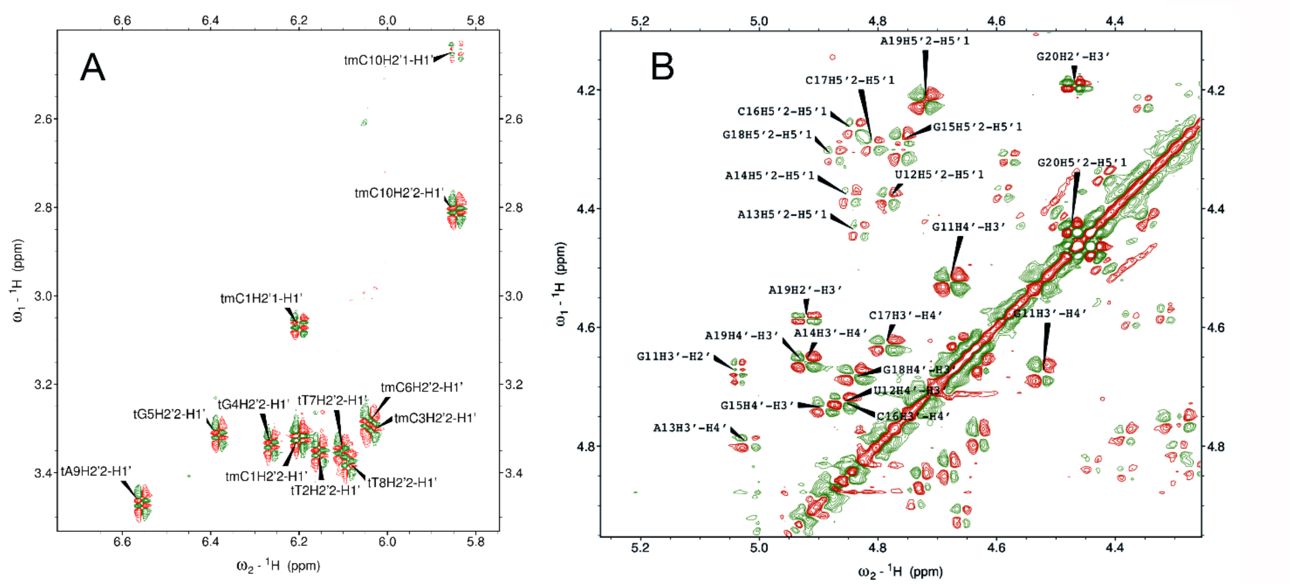

Figure S6. Expanded DQF-COSY spectrum of tc-DNA•RNA duplex, showing (A) strong H2'2 -H1' and very weak H2'1-H1' (peaks are missing) coupling constants of tc-DNA sugars and (B) strong H3'-4' and very weak H1'-H2' (peaks are missing) coupling constants of RNA sugars. The observed peaks are consistent with Northen sugar conformation.

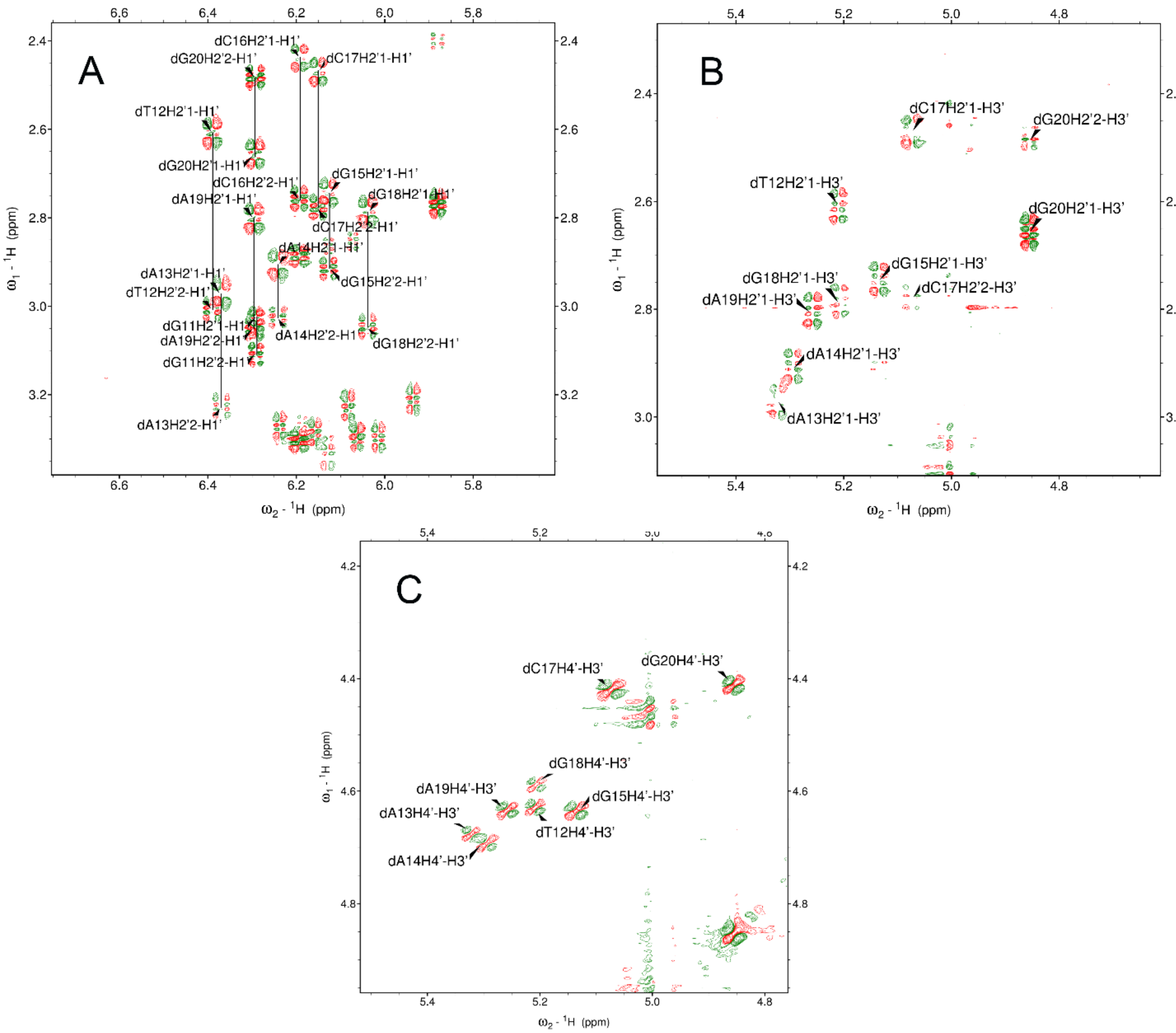

Figure S7. Expanded DQF-COSY spectrum of tc-DNA•DNA duplex, illustrating (A) strong H2'1 -H1', medium H2'2-H1', (B) undetectable H2'2-3' (peaks are missing) and (C) medium H4'-H3' coupling constants of DNA sugars. The observed peaks are consistent with the existence of a South-East-North deoxyribose conformation equilibrium.

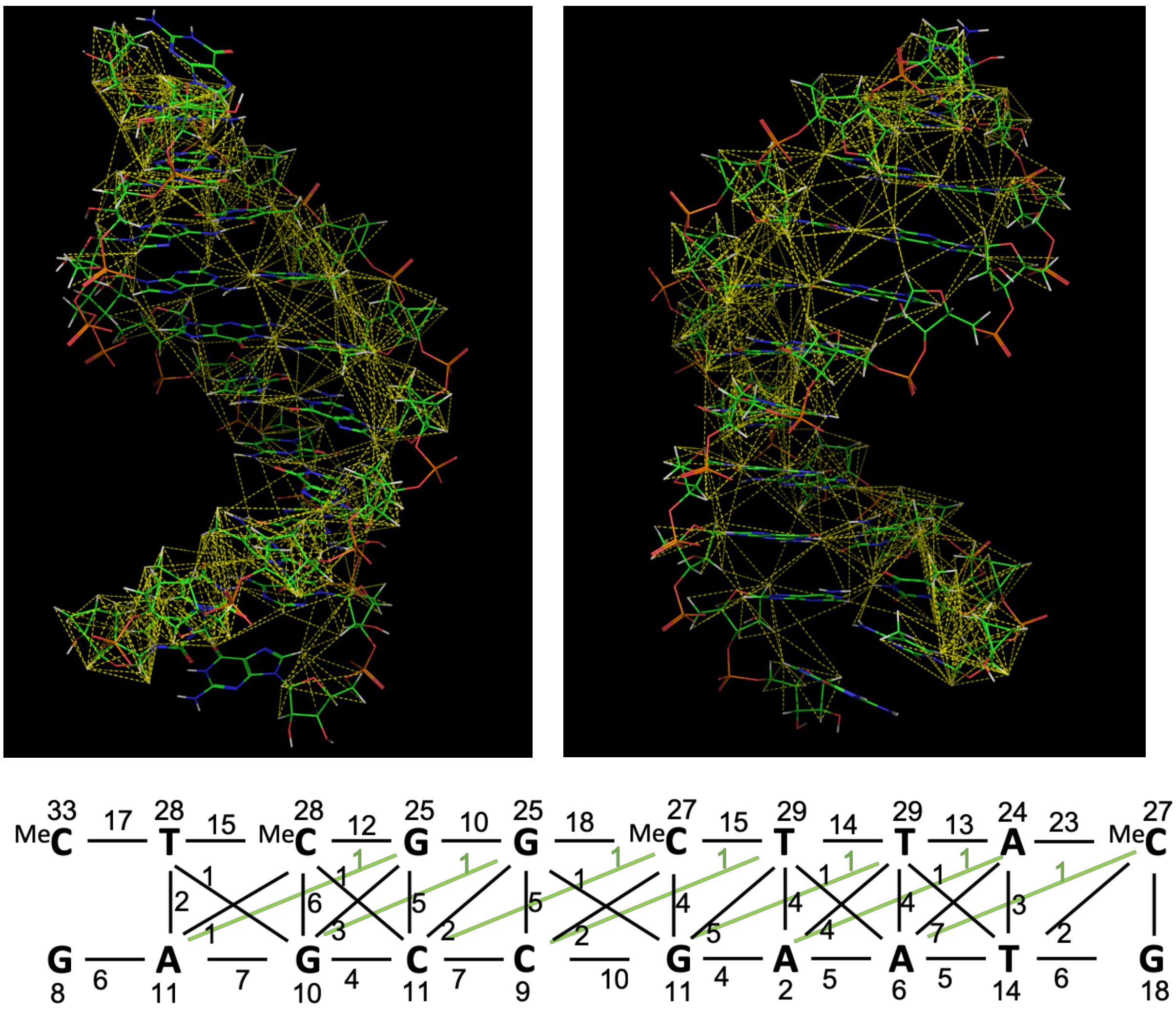

Figure S8. The distribution of the NOE restraints obtained from RANDMARDI calculations for Tc-DNA•RNA duplex

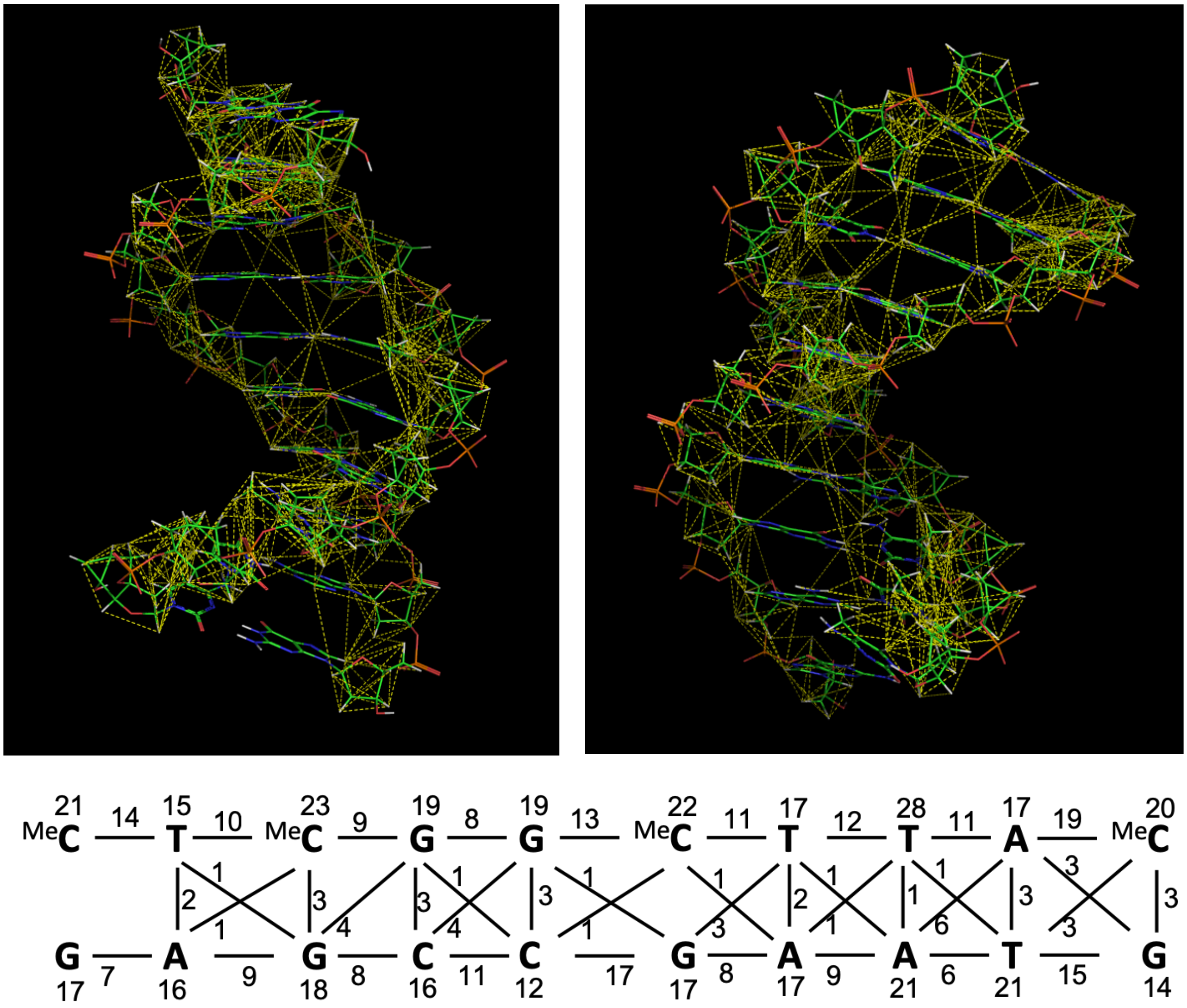

Figure S9. The distribution of the NOE restraints obtained from RANDMARDI calculations for Tc-DNA•DNA duplex.

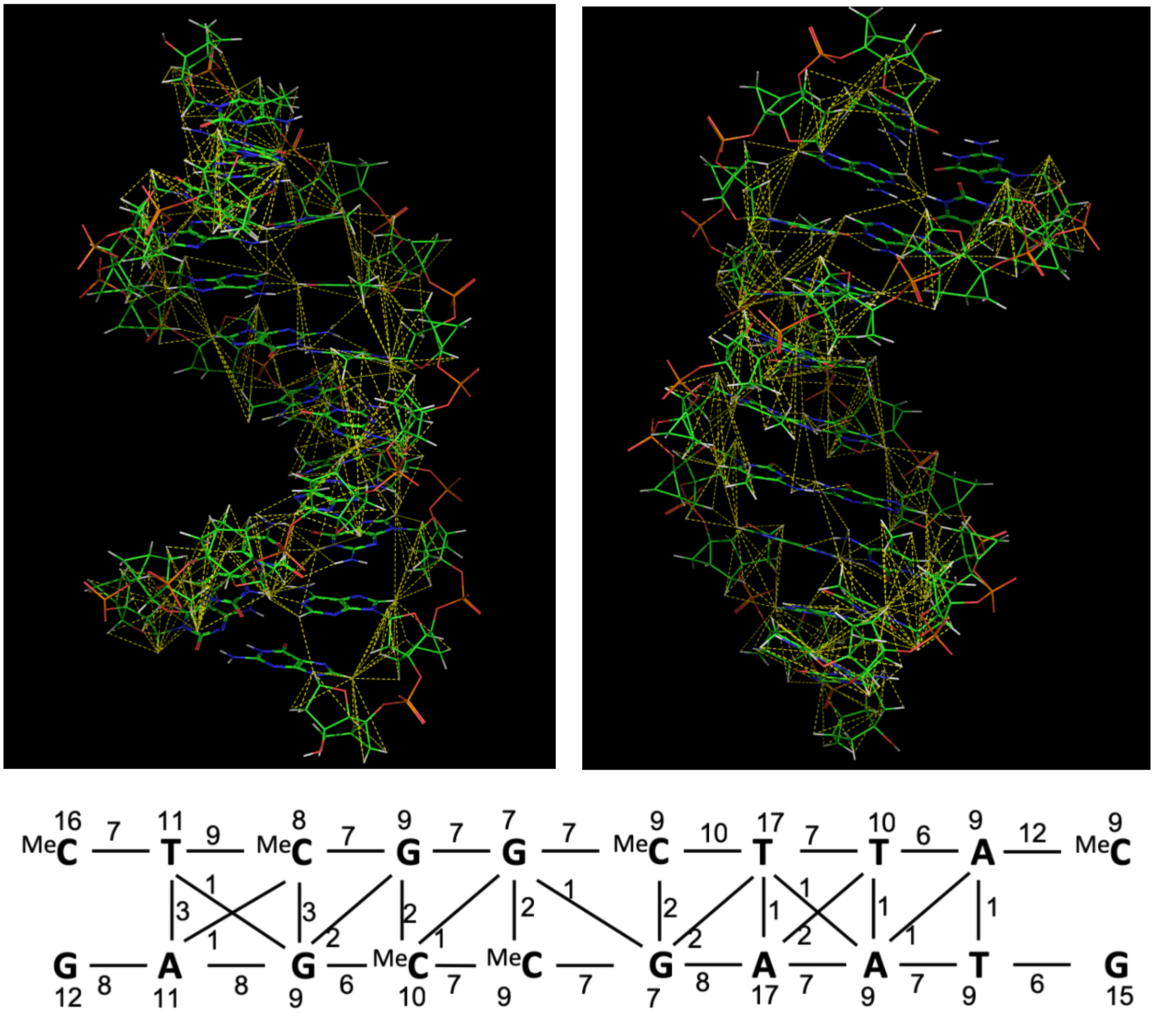

Figure S10. The distribution of the NOE restraints obtained from RANDMARDI calculations for tc-DNA•tc-DNA duplex.

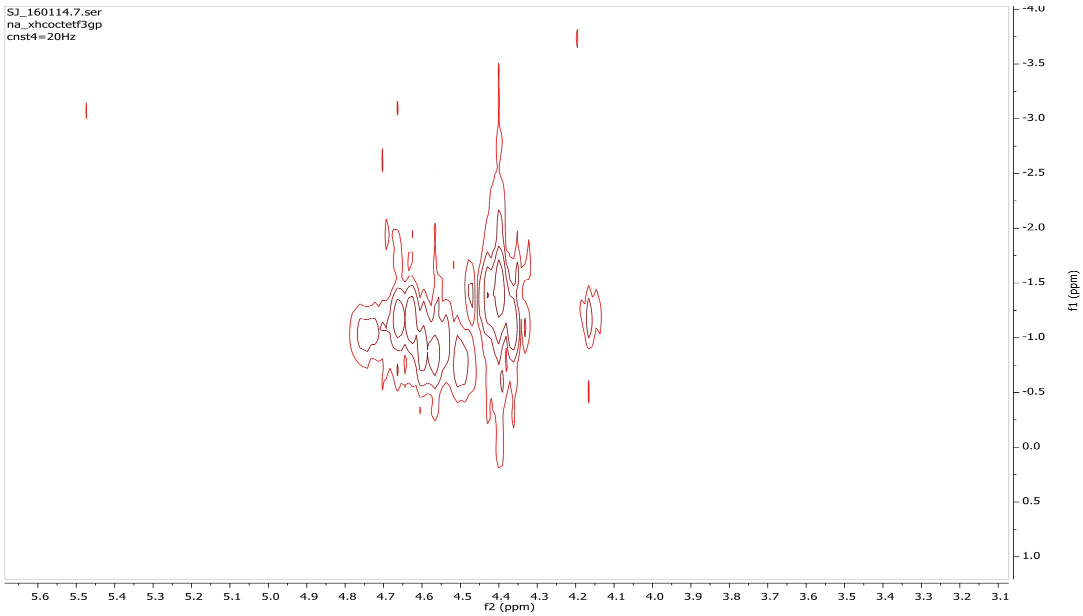

Figure S11. Expanded ^{1}H-^{31}P HETCOR spectrum of tc-DNA•RNA.

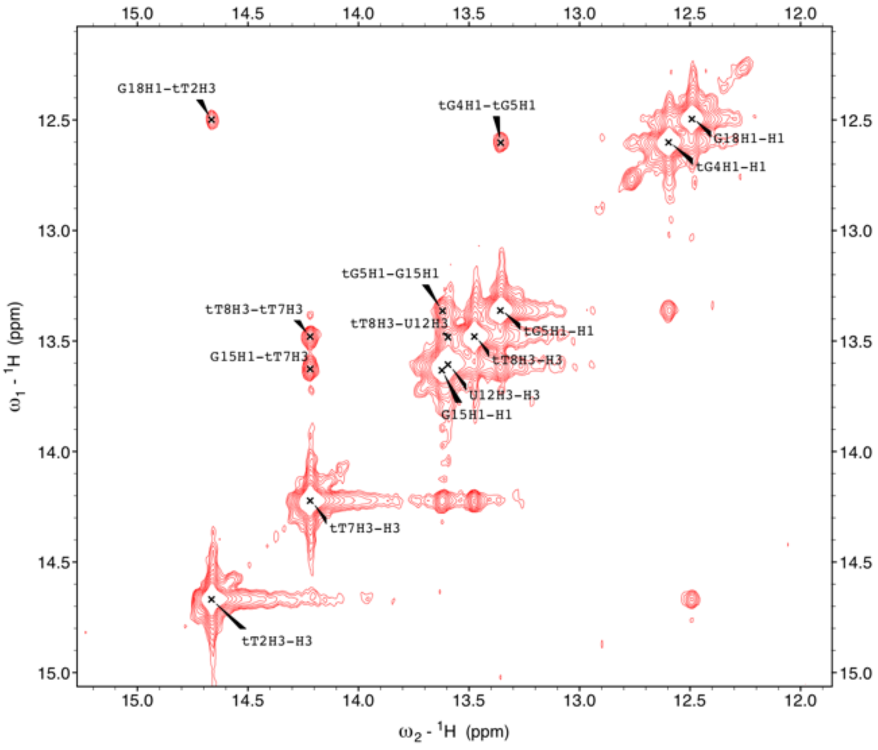

Figure S12. Diagonal imino region of the 250 ms NOESY spectrum of tc-DNA•RNA duplex recorded at 283 K.

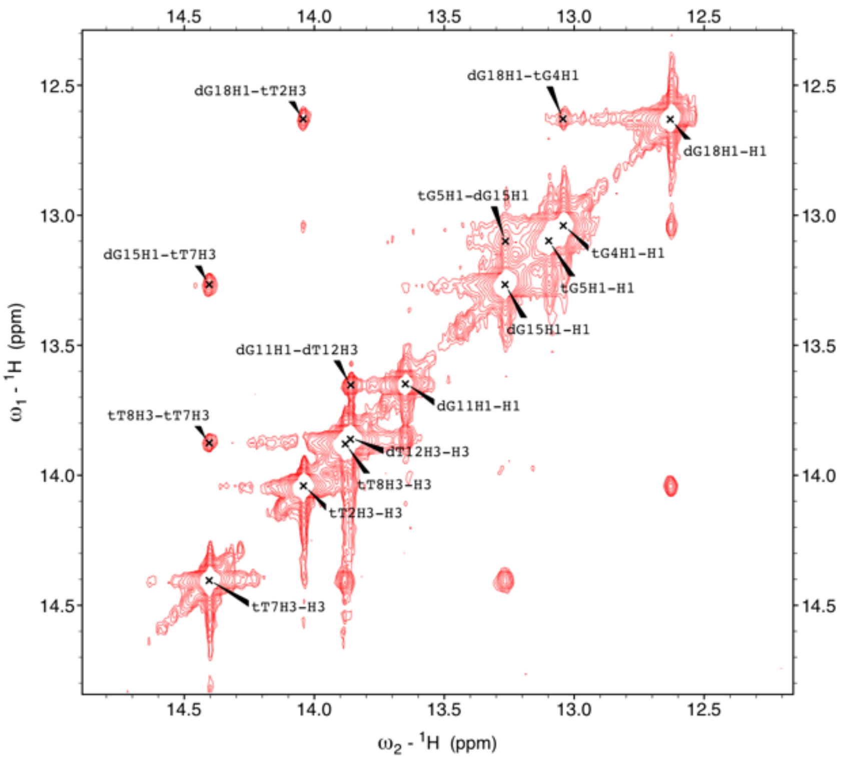

Figure S13. Diagonal imino region of the 250 ms NOESY spectrum of tc-DNA•DNA duplex recorded at 283 K.

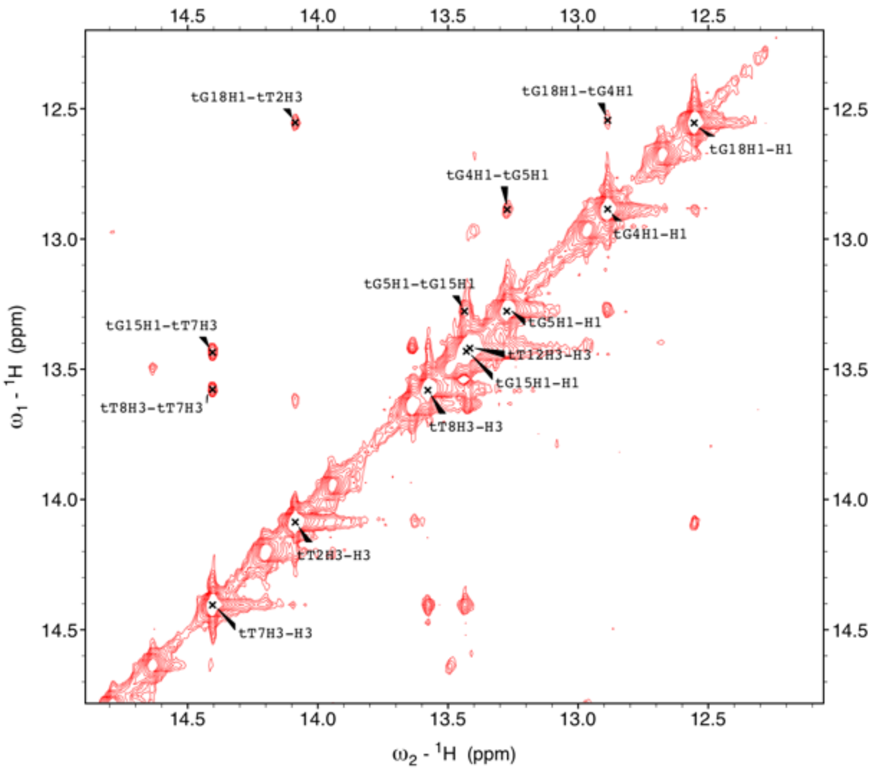

Figure S14. Diagonal imino region of the 250 ms NOESY spectrum of tc-DNA•tc-DNA duplex recorded at 283 K.

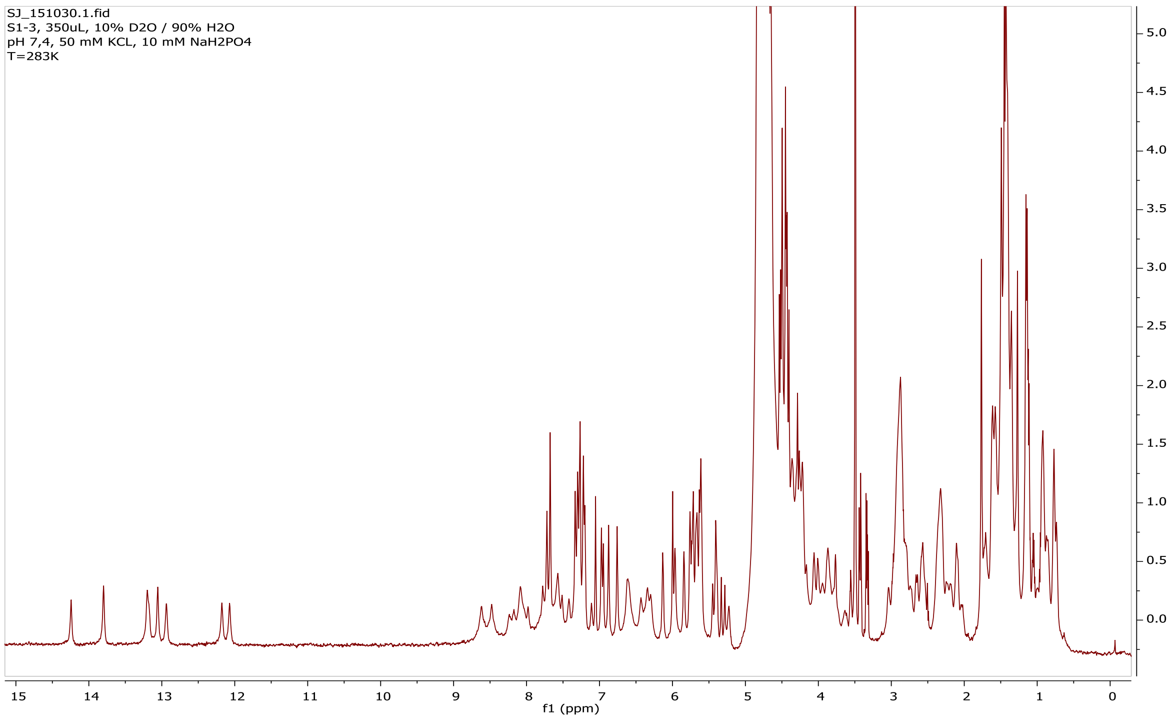

Figure S15. One-dimensional NMR spectrum of tc-DNA•RNA duplex in H_2O at 283 K.

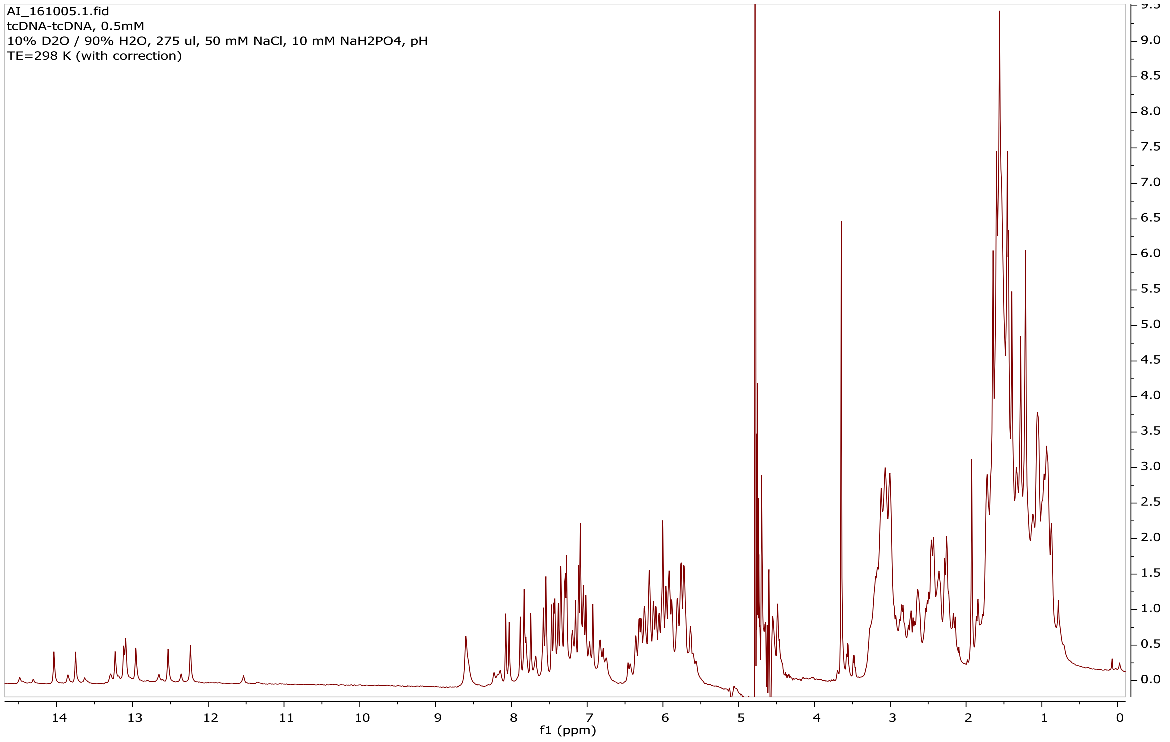

Figure S16. One-dimensional NMR spectrum of tc-DNA•tc-DNA duplex in H_2O at 283 K.

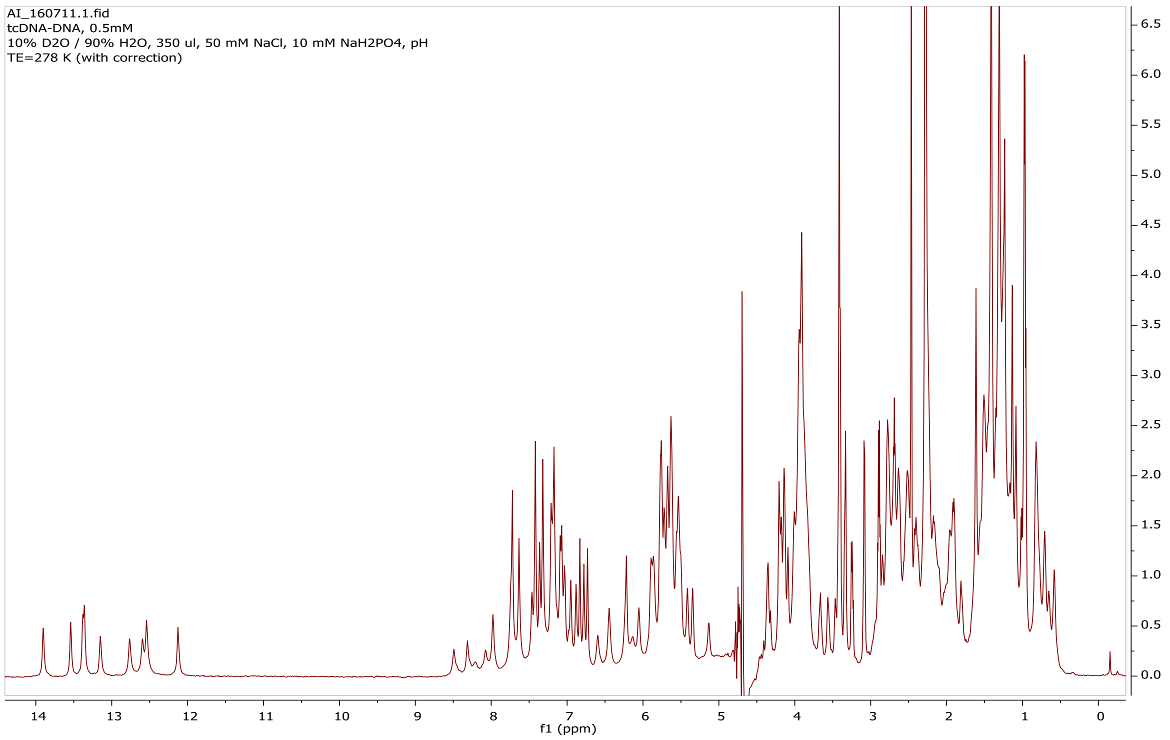

Figure S17. One-dimensional NMR spectrum of tc-DNA•DNA duplex in H_2O at 283 K.

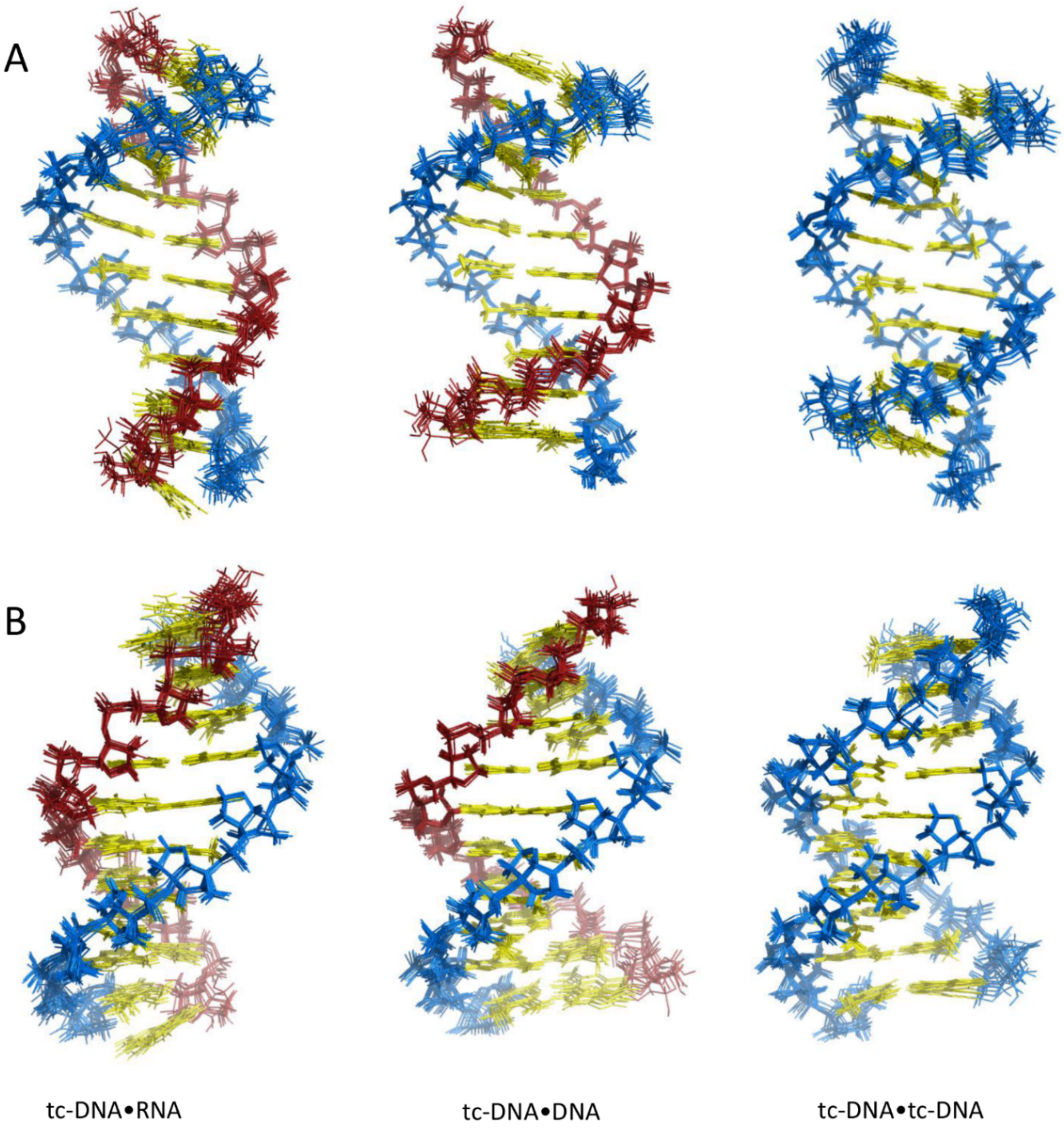

Figure S18. Comparison of the tc-DNA•RNA, tc-DNA•DNA, and tc-DNA•tc-DNA ensembles of 10 structures with lowest NOE energy. **(A)** View from the major groove. **(B)** View from the minor groove. The nucleobases are shown in yellow, tc-DNA sugar-phosphate backbone in blue and DNA/RNA sugar-phosphate backbone in red.

Table S1. Nonexchangeable ^{1}H chemical shifts for tc-DNA•RNA duplex at 25 °C.

Residue	H1'	H2	H2'	H2'1	H2'2	H3'	H4'	H5	H5'1	H5'2	H6	H6'	H7'1	H7'2	H7*	H8	H8'1	H8'2
tmC1	6.20	-	-	3.07	3.32	-	4.85	-	-	-	8.10	1.92	2.15	2.54	2.19	-	1.18	1.82
tT2	6.16	-	-	3.26	3.35	-	4.92	-	-	-	7.77	2.04	2.06	2.78	1.86	-	1.36	1.84
tmC3	6.04	-	-	2.98	3.30	-	4.92	-	-	-	7.66	1.99	2.03	2.62	1.92	-	1.34	1.79
tG4	6.27	-	-	3.22	3.34	-	4.88	-	-	-	-	1.84	1.57	2.54	-	7.70	1.20	1.78
tG5	6.38	-	-	3.23	3.32	-	4.88	-	-	-	-	1.78	1.45	2.73	-	7.40	1.21	1.83
tmC6	6.05	-	-	3.00	3.29	-	4.83	-	-	-	7.31	1.89	1.86	2.67	1.58	-	1.30	1.80
tT7	6.10	-	-	3.14	3.35	-	4.87	-	-	-	7.63	1.99	1.98	2.77	1.70	-	1.35	1.86
tT8	6.09	-	-	3.00	3.38	-	4.94	-	-	-	7.73	2.02	2.11	2.74	1.88	-	1.36	1.84
tA9	6.56	7.40	-	3.29	3.47	-	4.71	-	-	-	-	1.91	1.59	2.73	-	8.15	1.27	1.87
tmC10	5.85	-	-	2.45	2.81	-	4.71	-	-	-	7.20	1.83	1.86	1.93	1.55	-	1.18	1.73
G11	5.89	-	5.04	-	-	4.67	4.52	-	4.20	4.08	-	-	-	-	-	8.21	-	-
U12	5.83	-	4.68	-	-	4.86	4.73	5.35	4.79	4.38	8.08	-	-	-	-	-	-	-
A13	6.17	6.45	n.a.	-	-	4.79	5.01	-	4.84	4.44	-	-	-	-	-	8.39	-	-
A14	6.04	7.50	4.91	-	-	4.65	4.92	-	4.84	4.38	-	-	-	-	-	7.97	-	-
G15	5.83	-	4.67	-	-	4.89	4.73	-	4.77	4.28	-	-	-	-	-	7.50	-	-
C16	5.75	-	4.65	-	-	4.72	4.85.	5.28	4.85	4.27	7.85	-	-	-	-	-	-	-
C17	5.71	-	4.89	-	-	4.63	4.79	5.66	4.81	4.30	7.99	-	-	-	-	-	-	-
G18	5.87	-	4.78	-	-	4.84	4.68	-	4.88	4.31	-	-	-	-	-	7.72	-	-
A19	6.13	7.70	4.59	-	-	4.92	4.66	-	4.73	4.22	-	-	-	-	-	7.93	-	-
G20	6.05	-	4.24	-	-	4.47	4.73	-	4.47	4.43	-	-	-	-	-	7.63	-	-

n.a – not assigned.

Table S2. Exchangeable ^{1}H chemical shifts for tc-DNA•RNA duplex at 10 °C.

Residue	H1	H21	H22	H3	H41	H42	H61	H62
tmC1	-	-	-	-	8.6	7.05	-	-
tT2	-	-	-	14.66	-	-	-	-
tmC3	-	-	-	-	8.9	6.73	-	-
tG4	12.6	8.46	5.98	-	-	-	-	-
tG5	13.36	8.43	6.05	-	-	-	-	-
tmC6	-	-	-	-	9.04	6.77	-	-
tT7	-	-	-	14.22	-	-	-	-
tT8	-	-	-	13.48	-	-	-	-
tA9	-	-	-	-	-	-	8.19	6.39
tmC10	-	-	-	-	8.67	6.86	-	-
G11	13.28	n.a.	n.a.	-	-	-	-	-
U12	-	-	-	13.59	-	-	-	-
A13	-	-	-	-	-	-	8.04	6.81
A14	-	-	-	-	-	-	8.16	6.8
G15	13.62	n.a.	n.a.	-	-	-	-	-
C16	-	-	-	-	8.5	7.03	-	-
C17	-	-	-	-	8.51	7.02	-	-
G18	12.49	n.a.	n.a.	-	-	-	-	-
A19	-	-	-	-	-	-	8.60	7.05.
G20	n.a.	n.a.	n.a.	-	-	-	-	-

n.a – not assigned.

Table S3. ^{13}C chemical shifts for tc-DNA•RNA duplex at 25 °C.

Residue	C1'	C2	C2'	C3'	C4'	C5	C5'	C6	C6'	C7'	C8	C8'
tmC1	90.67	-	n.a.	n.a.	93.62	-	n.a.	140.7	25.64	42.28	-	18.42
tT2	91.96	-	n.a.	n.a.	93.4	-	n.a.	138.4	25.48	41.96	-	17.33
tmC3	91.74	-	n.a.	n.a.	93.39	-	n.a.	137.9	25.25	40.47	-	17.17
tG4	90.69	-	n.a.	n.a.	93.34	-	n.a.	-	25.69	41.06	136.3	17.17
tG5	90.89	-	n.a.	n.a.	93.15	-	n.a.	-	25.43	41.7	136.3	17.2
tmC6	90.83	-	n.a.	n.a.	92.8	-	n.a.	137.9	24.82	41.34	-	16.99
tT7	91.96	-	n.a.	n.a.	93.57	-	n.a.	137.9	25.69	41.86	-	17.23
tT8	91.92	-	n.a.	n.a.	93.42	-	n.a.	137.6	25.48	40.58	-	17.3
tA9	91.14	153.1	n.a.	n.a.	92.62	-	n.a.	-	25.64	42.33	139.8	17.3
tmC10	90.97	-	49.95	n.a.	91.88	-	n.a.	137.9	25.5	42.39	-	17.16
G11	93.25	-	74.97	72.67	84.73	-	n.a.	-	-	-	139	-
U12	93.95	-	74	72.63	n.a.	102.4	64.87	142.2	-	-	-	-
A13	92.94	151.9	n.a.	71.88	82.08	-	65.14	-	-	-	140	-
A14	92.73	153.3	75.46	n.a.	n.a.	-	64.8	-	-	-	-	-
G15	92.94	-	73.79	72.9	82.06	-	64.9	-	-	-	135.8	-
C16	94.11	-	75.14	n.a.	n.a.	96.55	64.21	141.1	-	-	-	-
C17	93.89	-	74.94	71.87	82.19	97.63	64.11	140.8	-	-	-	-
G18	93.16	-	75.08	72.56	82.03	-	64.3	-	-	-	136.3	-
A19	93.33	153.6	75.56	72.37	82.25	-	65.68	-	-	-	139.4	-
G20	-	-	76.44	70.58	n.a.	-	70.57	-	-	-	137.2	-

n.a – not assigned.

Table S4. Nonexchangeable ^{1}H chemical shifts for tc-DNA•DNA duplex at 25 °C.

Residue	H1'	H2	H2'1	H2'2	H3'	H4'	H5	H5'1	H5'2	H6	H6'	H7'1	H7'2	H7*	H8	H8'1	H8'2
tmC1	6.19	-	2.88	3.30	-	4.73	-	-	-	7.93	1.93	2.28	2.45	2.13	-	1.11	1.82
tT2	6.06	-	2.88	3.29	-	4.87	-	-	-	7.69	2.01	2.00	2.75	1.82	-	1.33	1.78
tmC3	6.08	-	3.18	3.22	-	4.91	-	-	-	7.62	2.02	2.10	2.63	1.92	-	1.33	1.78
tG4	6.16	-	3.21	3.29	-	4.88	-	-	-	-	1.92	1.72	2.54	-	7.71	1.22	1.78
tG5	6.24	-	3.14	3.27	-	4.86	-	-	-	-	1.92	1.54	2.67	-	7.38	1.22	1.78
tmC6	5.94	-	3.12	3.21	-	4.83	-	-	-	7.30	1.90	1.80	2.68	1.59	-	1.29	1.77
tT7	6.01	-	3.13	3.30	-	4.85	-	-	-	7.59	2.00	1.95	2.76	1.74	-	1.33	n.a.
tT8	6.13	-	3.13	3.34	-	4.94	-	-	-	7.74	2.06	2.16	2.76	1.93	-	1.34	1.82
tA9	6.57	7.39	3.27	3.46	-	n.a.	-	-	-	-	1.99	1.71	2.74	-	8.16	1.31	1.84
tmC10	5.88	-	2.40	2.77	-	4.72	-	-	-	7.26	1.84	n.a.	1.97	1.65	-	1.16	1.70
dG11	6.29	-	3.03	3.10	n.a.	4.47	-	4.16	4.06	-	-	-	-	-	8.23	-	-
dT12	6.39	-	2.61	3.01	5.21	4.63	-	4.42	4.42	7.82	-	-	-	1.51	-	-	-
dA13	6.37	6.75	2.97	3.23	5.32	4.68	-	4.38	4.52	-	-	-	-	-	8.45	-	-
dA14	6.24	7.48	2.91	3.03	5.29	4.69	-	4.51	4.51	-	-	-	-	-	8.20	-	-
dG15	6.13	-	2.75	2.92	5.14	4.63	-	4.47	4.47	-	-	-	-	-	7.78	-	-
dC16	6.19	-	2.44	2.76	n.a.	4.45	5.28	4.48	4.38	7.53	-	-	-	-	-	-	-
dC17	6.15	-	2.47	2.78	5.07	4.42	5.63	4.36	4.36	7.68	-	-	-	-	-	-	-
dG18	6.04	-	2.79	3.04	5.21	4.59	-	4.32	4.40	-	-	-	-	-	7.95	-	-
dA19	6.30	7.84	2.80	3.05	5.26	4.64	-	4.42	4.46	-	-	-	-	-	8.23	-	-
dG20	6.29	-	2.66	2.48	4.86	4.41	-	4.35	4.43	-	-	-	-	-	7.90	-	-

n.a – not assigned.

Table S5. Exchangeable ^{1}H chemical shifts for tc-DNA•DNA duplex at 10 °C.

Residue	H1	H3	H41	H42	H61	H62
tmC1	-	-	n.a.	n.a.	-	-
tT2	-	14.04	-	-	-	-
tmC3	-	-	8.991	6.737	-	-
tG4	13.04	-	-	-	-	-
tG5	13.1	-	-	-	-	-
tmC6	-	-	8.718	6.728	-	-
tT7	-	14.4	-	-	-	-
tT8	-	13.88	-	-	-	-
tA9	-	-	-	-	8.477	6.62
tmC10	-	-	8.815	6.943	-	-
dG11	13.65	-	-	-	-	-
dT12	-	13.86	-	-	-	-
dA13	-	-	-	-	8.135	6.489
dA14	-	-	-	-	8.023	6.439
dG15	13.27	-	-	-	-	-
dC16	-	-	8.229	6.635	-	-
dC17	-	-	8.578	6.947	-	-
dG18	12.63	-	-	-	-	-
dA19	-	-	-	-	8.207	6.345
tA19	-	-	-	-	n.a.	n.a.
dG20	n.a.	n.a.	-	-	-	-

n.a – not assigned.

Table S6. ^{13}C chemical shifts for tc-DNA•DNA duplex at 25 °C.

Residue	C1'	C2'	C3'	C4'	C5	C5'	C6'	C7'	C8'
tmC1	89.61	45.75	n.a.	92.96	n.a.	n.a.	25.8	42.52	18.97
tT2	91.88	n.a.	n.a.	n.a.	n.a.	n.a.	n.a.	n.a.	n.a.
tmC3	91.85	n.a.	n.a.	93.41	n.a.	n.a.	n.a.	40.83	n.a.
tG4	90.52	n.a.	n.a.	n.a.	n.a.	n.a.	n.a.	41.31	n.a.
tG5	90.67	n.a.	n.a.	n.a.	n.a.	n.a.	n.a.	n.a.	n.a.
tmC6	92.00	n.a.	n.a.	92.91	n.a.	n.a.	24.88	n.a.	n.a.
tT7	92.00	n.a.	n.a.	n.a.	n.a.	n.a.	n.a.	n.a.	n.a.
tT8	91.76	n.a.	n.a.	n.a.	n.a.	n.a.	n.a.	n.a.	n.a.
tA9	90.99	n.a.	n.a.	n.a.	n.a.	n.a.	n.a.	n.a.	n.a.
tmC10	90.80	49.88	n.a.	91.97	n.a.	n.a.	25.44	n.a.	n.a.
dG11	87.53	n.a.	n.a.	88.32	n.a.	63.15	-	-	-
dT12	86.27	n.a.	79.72	87.01	n.a.	n.a.	-	-	-
dA13	84.80	n.a.	79.39	87.09	n.a.	n.a.	-	-	-
dA14	84.23	n.a.	78.09	86.88	n.a.	68.09	-	-	-
dG15	84.85	n.a.	77.92	n.a.	n.a.	68.09	-	-	-
dC16	86.67	n.a.	n.a.	85.45	97.33	66.94	-	-	-
dC17	86.64	n.a.	77.09	n.a.	98.27	67.16	-	-	-
dG18	84.68	n.a.	79.97	87.10	n.a.	68.38	-	-	-
dA19	84.78	n.a.	78.64	86.65	n.a.	n.a.	-	-	-
dG20	84.44	n.a.	72.93	87.69	n.a.	n.a.	-	-	-

n.a – not assigned

Table S7. Nonexchangeable ^{1}H chemical shifts for tc-DNA•tc-DNA duplex at 25 °C.

Residue	H1'	H2	H2'1	H2'2	H4'	H6	H6'	H7'1	H7'2	H7*	H8	H8'1	H8'2
tmC1	6.25	-	3.01	3.34	4.85	8.12	1.95	2.15	2.56	2.22	-	1.20	1.85
tT2	6.17	-	3.38	3.35	n.a.	7.76	n.a.	2.03	2.77	1.85	-	n.a.	n.a.
tmC3	6.02	-	3.12	3.29	n.a.	7.65	n.a.	2.07	2.65	1.94	-	n.a.	n.a.
tG4	6.21	-	3.27	3.35	n.a.	-	n.a.	1.62	2.56	-	7.74	n.a.	n.a.
tG5	6.38	-	3.31	3.34	n.a.	-	n.a.	1.56	2.73	-	7.45	n.a.	n.a.
tmC6	6.06	-	3.05	3.30	4.86	7.35	n.a.	1.90	2.74	1.57	-	n.a.	n.a.
tT7	6.10	-	3.15	3.36	4.88	7.64	n.a.	1.98	2.81	1.73	-	1.35	2.01
tT8	6.02	-	2.93	3.37	n.a.	7.72	n.a.	2.12	2.74	1.89	-	n.a.	n.a.
tA9	6.57	7.37	3.29	3.46	n.a.	-	n.a.	1.61	2.73	-	8.17	n.a.	n.a.
tmC10	5.92	-	2.44	2.80	4.72	7.22	n.a.	2.44	2.80	1.51	-	n.a.	n.a.
tG11	6.47	-	3.31	3.41	4.93	-	1.91	1.75	2.53	-	8.37	1.16	1.68
tT12	6.20	-	3.50	3.41	n.a.	7.60	n.a.	2.15	2.70	1.69	-	n.a.	n.a.
tA13	6.54	6.29	3.38	3.57	n.a.	-	n.a.	1.76	2.66	-	8.32	n.a.	n.a.
tA14	6.47	7.40	3.41	3.47	n.a.	-	n.a.	1.67	2.57	-	7.87	n.a.	n.a.
tG15	6.34	-	3.29	3.35	n.a.	-	n.a.	1.61	2.77	-	7.39	n.a.	n.a.
tmC16	6.05	-	2.96	3.30	4.85	7.31	n.a.	1.94	2.75	1.51	-	n.a.	n.a.
tmC17	5.99	-	3.14	3.30	4.86	7.58	n.a.	2.08	2.65	1.75	-	n.a.	n.a.
tG18	6.29	-	3.23	3.42	n.a.	-	n.a.	1.76	2.64	-	7.84	n.a.	n.a.
tA19	6.60	7.56	3.43	3.50	n.a.	-	1.86	1.77	2.56	-	8.04	1.25	1.86
tG20	6.42	-	2.92	2.94	4.82	-	1.68	n.a.	n.a.	-	7.68	1.07	1.74

n.a – not assigned

Table S8. Exchangeable ^{1}H chemical shifts for tc-DNA•tc-DNA duplex at 10 °C.

Residue	H1	H21	H22	H3	H41	H42	H61	H62
tmC1	-	-	-	-	n.a.	n.a.	-	-
tT2	-	-	-	14.09	-	-	-	-
tmC3	-	-	-	-	8.901	6.744	-	-
tG4	12.89	n.a.	n.a.	-	-	-	-	-
tG5	13.27	n.a.	n.a.	-	-	-	-	-
tmC6	-	-	-	-	8.904	6.767	-	-
tT7	-	-	-	14.4	-	-	-	-
tT8	-	-	-	13.58	-	-	-	-
tA9	-	-	-	-	-	-	n.a.	n.a.
tmC10	-	-	-	-	n.a.	n.a.	-	-
tG11	n.a.	n.a.	n.a.	-	-	-	-	-
tT12	-	-	-	13.42	-	-	-	-
tA13	-	-	-	-	-	-	n.a.	n.a.
tA14	-	-	-	-	-	-	n.a.	n.a.
tG15	13.44	n.a.	n.a.	-	-	-	-	-
tmC16	-	-	-	-	8.893	6.753	-	-
tmC17	-	-	-	-	8.891	6.684	-	-
tG18	12.55	7.635	n.a.	-	-	-	-	-
tA19	-	-	-	-	-	-	8.489	6.484
tG20	n.a.	n.a.	n.a.	-	-	-	-	-

n.a – not assigned

AMBER forcefield parameters used for the modified nucleotides

The partial charges were derived from QM calculations at HF/6-31G* theory level with the GAUSSIAN 09 (1) and fitted to each atomic centre with R.E.D.-III.5 (2) tools program package according to the RESP (3) algorithm. Three new atom types were introduced to account for the three-membered ring in tricyclo-DNA: C8, C6, C5. The bond length angle values for the new atom types were derived from QM calculations at HF/6-31G* theory level with the GAUSSIAN 09. The Kr and K_0 were assigned by analogy with similar atom types. This is a good approximation for the restrained MD calculations performed in this research but might be not accurate enough for unrestrained MD calculations.

```
RESI TA  ! Tc-ADENOSINE
GROUP
ATOM    P    TYPE=P          CHARGE= 1.21660 END  !
ATOM   O1P   TYPE=O2         CHARGE=-0.79140 END  !
ATOM   O2P   TYPE=O2         CHARGE=-0.79140 END  !
ATOM   O5'   TYPE=OS         CHARGE=-0.49280 END  !
ATOM   C5'   TYPE=C5         CHARGE= 0.20640 END  !
ATOM   C4'   TYPE=CT         CHARGE= 0.19590 END  !
ATOM   H4'   TYPE=H1         CHARGE= 0.09370 END  !
ATOM   O4'   TYPE=OS         CHARGE=-0.45460 END  !
ATOM   C1'   TYPE=CT         CHARGE= 0.16710 END  !
ATOM   H1'   TYPE=H2         CHARGE= 0.09440 END  !
ATOM    N9   TYPE=N*         CHARGE=-0.01910 END  !
ATOM    C8   TYPE=CK         CHARGE= 0.19630 END  !
ATOM    H8   TYPE=H5         CHARGE= 0.14300 END  !
ATOM    N7   TYPE=NB         CHARGE=-0.59160 END  !
ATOM    C5   TYPE=CB         CHARGE= 0.02890 END  !
ATOM    C6   TYPE=CA         CHARGE= 0.67280 END  !
ATOM    N6   TYPE=N2         CHARGE=-0.87840 END  !
ATOM   H61   TYPE=H          CHARGE= 0.39800 END  !
ATOM   H62   TYPE=H          CHARGE= 0.39800 END  !
ATOM    N1   TYPE=NC         CHARGE=-0.75700 END  !
ATOM    C2   TYPE=CQ         CHARGE= 0.60020 END  !
ATOM    H2   TYPE=H5         CHARGE= 0.04740 END  !
ATOM    N3   TYPE=NC         CHARGE=-0.75770 END  !
ATOM    C4   TYPE=CB         CHARGE= 0.39800 END  !
ATOM   C3'   TYPE=CT         CHARGE= 0.27860 END  !
ATOM   C2'   TYPE=CT         CHARGE=-0.07600 END  !
ATOM  H2'1   TYPE=HC         CHARGE= 0.05590 END  !
ATOM  H2'2   TYPE=HC         CHARGE= 0.05590 END  !
ATOM   C7'   TYPE=CT         CHARGE=-0.10070 END  !
ATOM  H7'1   TYPE=HC         CHARGE= 0.04060 END  !
ATOM  H7'2   TYPE=HC         CHARGE= 0.04060 END  !
ATOM   C6'   TYPE=C6         CHARGE=-0.06740 END  !
ATOM   H6'   TYPE=HC         CHARGE= 0.12560 END  !
ATOM   C8'   TYPE=C8         CHARGE=-0.40430 END  !
ATOM  H8'1   TYPE=H8         CHARGE= 0.15500 END  !
ATOM  H8'2   TYPE=H8         CHARGE= 0.15500 END  !
ATOM   O3'   TYPE=OS         CHARGE=-0.57750 END  !
BOND    P   O1P
```

```
BOND    P   O2P
BOND    P   O5'
BOND   O5'   C5'
BOND   C5'   C4'
BOND   C5'   C6'
BOND   C5'   C8'
BOND   C4'   H4'
BOND   C4'   O4'
BOND   C4'   C3'
BOND   O4'   C1'
BOND   C1'   H1'
BOND   C1'   N9
BOND   C1'   C2'
BOND   N9   C8
BOND   N9   C4
BOND   C8   H8
BOND   C8   N7
BOND   N7   C5
BOND   C5   C6
BOND   C5   C4
BOND   C6   N6
BOND   C6   N1
BOND   N6   H61
BOND   N6   H62
BOND   N1   C2
BOND   C2   H2
BOND   C2   N3
BOND   N3   C4
BOND   C3'   C2'
BOND   C3'   O3'
BOND   C3'   C7'
BOND   C2'  H2'1
BOND   C2'  H2'2
BOND   C7'  H7'1
BOND   C7'  H7'2
BOND   C7'  C6'
BOND   C6'  H6'
BOND   C6'  C8'
BOND   C8'  H8'1
BOND   C8'  H8'2
IMPR C4   C8   N9   C1'
IMPR N9   N7   C8   H8
IMPR C5   N1   C6   N6
IMPR N1   N3   C2   H2
IMPR C6   H61  N6   H62
! IMPRoper to keep the two purine rings parallel:
IMPR C8   C4   C5   N1
IMPR N3   C4   C5   N7
IMPR C8   C5   C4   C2
IMPR C6   C5   C4   N9
! Other
```

```
IMPRoper N9   C4   C5   N7
IMPRoper C5   N7   C8   N9
IMPRoper C8   N9   C4   C5
IMPRoper C5   C6   N6   H61
END

RESI TT ! Tc-THYMINE
ATOM     P    TYPE=P          CHARGE= 1.21660 END  !
ATOM   O1P    TYPE=O2         CHARGE=-0.79140 END  !
ATOM   O2P    TYPE=O2         CHARGE=-0.79140 END  !
ATOM   O5'    TYPE=OS         CHARGE=-0.49280 END  !
ATOM   C5'    TYPE=C5         CHARGE= 0.20640 END  !
ATOM   C4'    TYPE=CT         CHARGE= 0.19590 END  !
ATOM   H4'    TYPE=H1         CHARGE= 0.09370 END  !
ATOM   O4'    TYPE=OS         CHARGE=-0.45860 END  !
ATOM   C1'    TYPE=CT         CHARGE= 0.19280 END  !
ATOM   H1'    TYPE=H2         CHARGE= 0.09620 END  !
ATOM    N1    TYPE=N*         CHARGE= 0.00980 END  !
ATOM    C6    TYPE=CM         CHARGE=-0.20290 END  !
ATOM    H6    TYPE=H4         CHARGE= 0.21610 END  !
ATOM    C5    TYPE=CM         CHARGE=-0.01550 END  !
ATOM    C7    TYPE=CT         CHARGE=-0.27030 END  !
ATOM   H71    TYPE=HC         CHARGE= 0.09290 END  !
ATOM   H72    TYPE=HC         CHARGE= 0.09290 END  !
ATOM   H73    TYPE=HC         CHARGE= 0.09290 END  !
ATOM    C4    TYPE=C          CHARGE= 0.53960 END  !
ATOM    O4    TYPE=O          CHARGE=-0.54300 END  !
ATOM    N3    TYPE=NA         CHARGE=-0.45100 END  !
ATOM    H3    TYPE=H          CHARGE= 0.34060 END  !
ATOM    C2    TYPE=C          CHARGE= 0.54240 END  !
ATOM    O2    TYPE=O          CHARGE=-0.59320 END  !
ATOM   C3'    TYPE=CT         CHARGE= 0.27860 END  !
ATOM   C2'    TYPE=CT         CHARGE=-0.07600 END  !
ATOM  H2'1    TYPE=HC         CHARGE= 0.05590 END  !
ATOM  H2'2    TYPE=HC         CHARGE= 0.05590 END  !
ATOM   C7'    TYPE=CT         CHARGE=-0.10070 END  !
ATOM  H7'1    TYPE=HC         CHARGE= 0.04060 END  !
ATOM  H7'2    TYPE=HC         CHARGE= 0.04060 END  !
ATOM   C6'    TYPE=C6         CHARGE=-0.06740 END  !
ATOM   H6'    TYPE=HC         CHARGE= 0.12560 END  !
ATOM   C8'    TYPE=C8         CHARGE=-0.40430 END  !
ATOM  H8'1    TYPE=H8         CHARGE= 0.15500 END  !
ATOM  H8'2    TYPE=H8         CHARGE= 0.15500 END  !
ATOM   O3'    TYPE=OS         CHARGE=-0.57750 END  !
BOND     P   O1P
BOND     P   O2P
BOND     P   O5'
BOND   O5'   C5'
BOND   C5'   C4'
BOND   C5'   C6'
BOND   C5'   C8'
```

```
BOND   C4'   H4'
BOND   C4'   O4'
BOND   C4'   C3'
BOND   O4'   C1'
BOND   C1'   H1'
BOND   C1'   N1
BOND   C1'   C2'
BOND   N1    C6
BOND   N1    C2
BOND   C6    H6
BOND   C6    C5
BOND   C5    C7
BOND   C5    C4
BOND   C7    H71
BOND   C7    H72
BOND   C7    H73
BOND   C4    O4
BOND   C4    N3
BOND   N3    H3
BOND   N3    C2
BOND   C2    O2
BOND   C3'   C2'
BOND   C3'   O3'
BOND   C3'   C7'
BOND   C2'  H2'1
BOND   C2'  H2'2
BOND   C7'  H7'1
BOND   C7'  H7'2
BOND   C7'  C6'
BOND   C6'  H6'
BOND   C6'  C8'
BOND   C8'  H8'1
BOND   C8'  H8'2
IMPR    C2    C6    N1    C1'
IMPR    C4    C6    C5    C7
IMPR    N1    N3    C2    O2
IMPR    C5    N3    C4    O4
IMPR    C4    C2    N3    H3
IMPR    N1    C5    C6    H6
IMPR    N1    C6    C5    C7
!other
IMPRoper C1'  C2   C6   N1
IMPRoper C4   C5   C6   N1
IMPRoper N1   C2   N3   C4
IMPRoper C6   N1   C2   N3
END

RESI TG  ! Tc-GUANINE
ATOM       P    TYPE=P          CHARGE= 1.21660 END  !
ATOM     O1P    TYPE=O2         CHARGE=-0.79140 END  !
ATOM     O2P    TYPE=O2         CHARGE=-0.79140 END  !
```

```
ATOM    O5'   TYPE=OS        CHARGE=-0.49280 END  !
ATOM    C5'   TYPE=C5        CHARGE= 0.20640 END  !
ATOM    C4'   TYPE=CT        CHARGE= 0.19590 END  !
ATOM    H4'   TYPE=H1        CHARGE= 0.09370 END  !
ATOM    O4'   TYPE=OS        CHARGE=-0.45860 END  !
ATOM    C1'   TYPE=CT        CHARGE= 0.12510 END  !
ATOM    H1'   TYPE=H2        CHARGE= 0.12920  END  !
ATOM     N9   TYPE=N*        CHARGE= 0.00540 END  !
ATOM     C8   TYPE=CK        CHARGE= 0.17740 END  !
ATOM     H8   TYPE=H5        CHARGE= 0.15680 END  !
ATOM     N7   TYPE=NB        CHARGE=-0.58030 END  !
ATOM     C5   TYPE=CB        CHARGE= 0.14320 END  !
ATOM     C6   TYPE=C         CHARGE= 0.55740 END  !
ATOM     O6   TYPE=O         CHARGE=-0.54950 END  !
ATOM     N1   TYPE=NA        CHARGE=-0.58840 END  !
ATOM     H1   TYPE=H         CHARGE= 0.36700 END  !
ATOM     C2   TYPE=CA        CHARGE= 0.79380 END  !
ATOM     N2   TYPE=N2        CHARGE=-0.91350 END  !
ATOM    H21   TYPE=H         CHARGE= 0.39740 END  !
ATOM    H22   TYPE=H         CHARGE= 0.39740 END  !
ATOM     N3   TYPE=NC        CHARGE=-0.68500 END  !
ATOM     C4   TYPE=CB        CHARGE= 0.20690 END  !
ATOM    C3'   TYPE=CT        CHARGE= 0.27860 END  !
ATOM    C2'   TYPE=CT        CHARGE=-0.07600 END  !
ATOM   H2'1   TYPE=HC        CHARGE= 0.05590 END  !
ATOM   H2'2   TYPE=HC        CHARGE= 0.05590 END  !
ATOM    C7'   TYPE=CT        CHARGE=-0.10070 END  !
ATOM   H7'1   TYPE=HC        CHARGE= 0.04060 END  !
ATOM   H7'2   TYPE=HC        CHARGE= 0.04060 END  !
ATOM    C6'   TYPE=C6        CHARGE=-0.06740 END  !
ATOM    H6'   TYPE=HC        CHARGE= 0.12560 END  !
ATOM    C8'   TYPE=C8        CHARGE=-0.40430 END  !
ATOM   H8'1   TYPE=H8        CHARGE= 0.15500 END  !
ATOM   H8'2   TYPE=H8        CHARGE= 0.15500 END  !
ATOM    O3'   TYPE=OS        CHARGE=-0.57750 END  !
BOND     P   O1P
BOND     P   O2P
BOND     P   O5'
BOND    O5'  C5'
BOND    C5'  C4'
BOND    C5'  C6'
BOND    C5'  C8'
BOND    C4'  H4'
BOND    C4'  O4'
BOND    C4'  C3'
BOND    O4'  C1'
BOND    C1'  H1'
BOND    C1'   N9
BOND    C1'  C2'
BOND     N9   C8
BOND     N9   C4
```

```
BOND     C8    H8
BOND     C8    N7
BOND     N7    C5
BOND     C5    C6
BOND     C5    C4
BOND     C6    O6
BOND     C6    N1
BOND     N1    H1
BOND     N1    C2
BOND     C2    N2
BOND     C2    N3
BOND     N2   H21
BOND     N2   H22
BOND     N3    C4
BOND     C3'   C2'
BOND     C3'   O3'
BOND     C3'   C7'
BOND     C2'  H2'1
BOND     C2'  H2'2
BOND     C7'  H7'1
BOND     C7'  H7'2
BOND     C7'  C6'
BOND     C6'  H6'
BOND     C6'  C8'
BOND     C8'  H8'1
BOND     C8'  H8'2
IMPR     C4    C8    N9   C1'
IMPR     C5    N1    C6    O6
IMPR     C6    C2    N1    H1
IMPR     C2   H21    N2   H22
IMPR     N9    N7    C8    H8
IMPR     N2    N1    C2    N3
!IMPRoper to keep the two purine rings parallel:
IMPR C8   C4   C5   N1
IMPR C8   C5   C4   C2
IMPR N3   C4   C5   N7
IMPR C6   C5   C4   N9
!other
IMPRoper N3   C2   N2   H21
IMPRoper N1   C2   N2   H22
IMPRoper N3   C2   N1   H1
IMPRoper N9   C4   C5   N7
IMPRoper C5   N7   C8   N9
IMPRoper C8   N9   C4   C5
END


RESI TG5  ! Tc-GUANINE 5’ terminal
ATOM   H5T    TYPE=HO         CHARGE= 0.42470 END  !
ATOM   O5'    TYPE=OH         CHARGE= -0.60250 END  !
```

```
ATOM    C5'   TYPE=C5         CHARGE= 0.20640 END  !
ATOM    C4'   TYPE=CT         CHARGE= 0.19590 END  !
ATOM    H4'   TYPE=H1         CHARGE= 0.09370 END  !
ATOM    O4'   TYPE=OS         CHARGE=-0.45860 END  !
ATOM    C1'   TYPE=CT         CHARGE= 0.12510 END  !
ATOM    H1'   TYPE=H2         CHARGE= 0.12920  END  !
ATOM     N9   TYPE=N*         CHARGE= 0.00540 END  !
ATOM     C8   TYPE=CK         CHARGE= 0.17740 END  !
ATOM     H8   TYPE=H5         CHARGE= 0.15680 END  !
ATOM     N7   TYPE=NB         CHARGE=-0.58030 END  !
ATOM     C5   TYPE=CB         CHARGE= 0.14320 END  !
ATOM     C6   TYPE=C          CHARGE= 0.55740 END  !
ATOM     O6   TYPE=O          CHARGE=-0.54950 END  !
ATOM     N1   TYPE=NA         CHARGE=-0.58840 END  !
ATOM     H1   TYPE=H          CHARGE= 0.36700 END  !
ATOM     C2   TYPE=CA         CHARGE= 0.79380 END  !
ATOM     N2   TYPE=N2         CHARGE=-0.91350 END  !
ATOM    H21    TYPE=H         CHARGE= 0.39740 END  !
ATOM    H22    TYPE=H         CHARGE= 0.39740 END  !
ATOM     N3   TYPE=NC         CHARGE=-0.68500 END  !
ATOM     C4   TYPE=CB         CHARGE= 0.20690 END  !
ATOM    C3'   TYPE=CT         CHARGE= 0.27860 END  !
ATOM    C2'   TYPE=CT         CHARGE=-0.07600 END  !
ATOM   H2'1    TYPE=HC         CHARGE= 0.05590 END  !
ATOM   H2'2    TYPE=HC         CHARGE= 0.05590 END  !
ATOM    C7'   TYPE=CT         CHARGE=-0.10070 END  !
ATOM   H7'1    TYPE=HC         CHARGE= 0.04060 END  !
ATOM   H7'2    TYPE=HC         CHARGE= 0.04060 END  !
ATOM    C6'   TYPE=C6         CHARGE=-0.06740 END  !
ATOM    H6'   TYPE=HC         CHARGE= 0.12560 END  !
ATOM    C8'   TYPE=C8         CHARGE=-0.40430 END  !
ATOM   H8'1    TYPE=H8         CHARGE= 0.15500 END  !
ATOM   H8'2    TYPE=H8         CHARGE= 0.15500 END  !
ATOM    O3'   TYPE=OS         CHARGE=-0.57750 END  !
BOND    H5T   O5'
BOND    O5'   C5'
BOND    C5'   C4'
BOND    C5'   C6'
BOND    C5'   C8'
BOND    C4'   H4'
BOND    C4'   O4'
BOND    C4'   C3'
BOND    O4'   C1'
BOND    C1'   H1'
BOND    C1'    N9
BOND    C1'   C2'
BOND     N9    C8
BOND     N9    C4
BOND     C8    H8
BOND     C8    N7
BOND     N7    C5
```

```
BOND     C5    C6
BOND     C5    C4
BOND     C6    O6
BOND     C6    N1
BOND     N1    H1
BOND     N1    C2
BOND     C2    N2
BOND     C2    N3
BOND     N2   H21
BOND     N2   H22
BOND     N3    C4
BOND     C3'  C2'
BOND     C3'  O3'
BOND     C3'  C7'
BOND     C2'  H2'1
BOND     C2'  H2'2
BOND     C7'  H7'1
BOND     C7'  H7'2
BOND     C7'  C6'
BOND     C6'  H6'
BOND     C6'  C8'
BOND     C8'  H8'1
BOND     C8'  H8'2
IMPR     C4    C8    N9   C1'
IMPR     C5    N1    C6    O6
IMPR     C6    C2    N1    H1
IMPR     C2   H21    N2   H22
IMPR     N9    N7    C8    H8
IMPR     N2    N1    C2    N3
!IMPRoper to keep the two purine rings parallel:
IMPR C8   C4   C5   N1
IMPR C8   C5   C4   C2
IMPR N3   C4   C5   N7
IMPR C6   C5   C4   N9
!other
IMPRoper N3   C2   N2   H21
IMPRoper N1   C2   N2   H22
IMPRoper N3   C2   N1   H1
IMPRoper N9   C4   C5   N7
IMPRoper C5   N7   C8   N9
IMPRoper C8   N9   C4   C5
END


RESI TG3  ! Tc-GUANINE
ATOM       P    TYPE=P           CHARGE= 1.21660 END  !
ATOM     O1P    TYPE=O2          CHARGE=-0.79140 END  !
ATOM     O2P    TYPE=O2          CHARGE=-0.79140 END  !
ATOM     O5'    TYPE=OS          CHARGE=-0.49280 END  !
ATOM     C5'    TYPE=C5          CHARGE= 0.20640 END  !
ATOM     C4'    TYPE=CT          CHARGE= 0.19590 END  !
```

```
ATOM     H4'    TYPE=H1           CHARGE= 0.09370 END  !
ATOM     O4'    TYPE=OS           CHARGE=-0.45860 END  !
ATOM     C1'    TYPE=CT           CHARGE= 0.12510 END  !
ATOM     H1'    TYPE=H2           CHARGE= 0.12920  END  !
ATOM      N9    TYPE=N*           CHARGE= 0.00540 END  !
ATOM      C8    TYPE=CK           CHARGE= 0.17740 END  !
ATOM      H8    TYPE=H5           CHARGE= 0.15680 END  !
ATOM      N7    TYPE=NB           CHARGE=-0.58030 END  !
ATOM      C5    TYPE=CB           CHARGE= 0.14320 END  !
ATOM      C6    TYPE=C            CHARGE= 0.55740 END  !
ATOM      O6    TYPE=O            CHARGE=-0.54950 END  !
ATOM      N1    TYPE=NA           CHARGE=-0.58840 END  !
ATOM      H1    TYPE=H            CHARGE= 0.36700 END  !
ATOM      C2    TYPE=CA           CHARGE= 0.79380 END  !
ATOM      N2    TYPE=N2           CHARGE=-0.91350 END  !
ATOM     H21    TYPE=H            CHARGE= 0.39740 END  !
ATOM     H22    TYPE=H            CHARGE= 0.39740 END  !
ATOM      N3    TYPE=NC           CHARGE=-0.68500 END  !
ATOM      C4    TYPE=CB           CHARGE= 0.20690 END  !
ATOM     C3'    TYPE=CT           CHARGE= 0.27860 END  !
ATOM     C2'    TYPE=CT           CHARGE=-0.07600 END  !
ATOM    H2'1    TYPE=HC           CHARGE= 0.05590 END  !
ATOM    H2'2    TYPE=HC           CHARGE= 0.05590 END  !
ATOM     C7'    TYPE=CT           CHARGE=-0.10070 END  !
ATOM    H7'1    TYPE=HC           CHARGE= 0.04060 END  !
ATOM    H7'2    TYPE=HC           CHARGE= 0.04060 END  !
ATOM     C6'    TYPE=C6           CHARGE=-0.06740 END  !
ATOM     H6'    TYPE=HC           CHARGE= 0.12560 END  !
ATOM     C8'    TYPE=C8           CHARGE=-0.40430 END  !
ATOM    H8'1    TYPE=H8           CHARGE= 0.15500 END  !
ATOM    H8'2    TYPE=H8           CHARGE= 0.15500 END  !
ATOM   O3'    TYPE=OH           CHARGE= -0.57750 END  !
ATOM  H3T     TYPE=HO           CHARGE=  0.44190 END  !
BOND       P   O1P
BOND       P   O2P
BOND       P   O5'
BOND     O5'   C5'
BOND     C5'   C4'
BOND     C5'   C6'
BOND     C5'   C8'
BOND     C4'   H4'
BOND     C4'   O4'
BOND     C4'   C3'
BOND     O4'   C1'
BOND     C1'   H1'
BOND     C1'    N9
BOND     C1'   C2'
BOND      N9    C8
BOND      N9    C4
BOND      C8    H8
BOND      C8    N7
```

```
BOND     N7    C5
BOND     C5    C6
BOND     C5    C4
BOND     C6    O6
BOND     C6    N1
BOND     N1    H1
BOND     N1    C2
BOND     C2    N2
BOND     C2    N3
BOND     N2   H21
BOND     N2   H22
BOND     N3    C4
BOND     C3'   C2'
BOND     C3'   O3'
BOND     C3'   C7'
BOND     C2'  H2'1
BOND     C2'  H2'2
BOND     C7'  H7'1
BOND     C7'  H7'2
BOND     C7'  C6'
BOND     C6'  H6'
BOND     C6'  C8'
BOND     C8'  H8'1
BOND     C8'  H8'2
BOND   O3'  H3T
IMPR     C4    C8    N9   C1'
IMPR     C5    N1    C6    O6
IMPR     C6    C2    N1    H1
IMPR     C2   H21    N2   H22
IMPR     N9    N7    C8    H8
IMPR     N2    N1    C2    N3
!IMPRoper to keep the two purine rings parallel:
IMPR C8   C4   C5   N1
IMPR C8   C5   C4   C2
IMPR N3   C4   C5   N7
IMPR C6   C5   C4   N9
!other
IMPRoper N3   C2   N2   H21
IMPRoper N1   C2   N2   H22
IMPRoper N3   C2   N1   H1
IMPRoper N9   C4   C5   N7
IMPRoper C5   N7   C8   N9
IMPRoper C8   N9   C4   C5
END


RESI TC  ! Tc-CYTOSINE
ATOM     P    TYPE=P          CHARGE= 1.16590 END  !
ATOM   O1P    TYPE=O2         CHARGE=-0.77610 END  !
ATOM   O2P    TYPE=O2         CHARGE=-0.77610 END  !
ATOM   O5'    TYPE=OS         CHARGE=-0.49280 END  !
```

```
ATOM   C5'   TYPE=C5         CHARGE= 0.19720 END  !
ATOM   C4'   TYPE=CT         CHARGE= 0.17180 END  !
ATOM   H4'   TYPE=H1         CHARGE= 0.09000 END  !
ATOM   O4'   TYPE=OS         CHARGE=-0.43510 END  !
ATOM   C1'   TYPE=CT         CHARGE= 0.17170 END  !
ATOM   H1'   TYPE=H2         CHARGE= 0.11130 END  !
ATOM    N1   TYPE=N*         CHARGE=-0.03390 END  !
ATOM    C6   TYPE=CM         CHARGE=-0.01830 END  !
ATOM    H6   TYPE=H4         CHARGE= 0.22930 END  !
ATOM    C5   TYPE=CM         CHARGE=-0.52220 END  !
ATOM    H5   TYPE=HA         CHARGE= 0.18630 END  !
ATOM    C4   TYPE=CA         CHARGE= 0.84390 END  !
ATOM    N4   TYPE=N2         CHARGE=-0.97730 END  !
ATOM   H41   TYPE=H          CHARGE= 0.43140 END  !
ATOM   H42   TYPE=H          CHARGE= 0.43140 END  !
ATOM    N3   TYPE=NC         CHARGE=-0.77480 END  !
ATOM    C2   TYPE=C          CHARGE= 0.79590 END  !
ATOM    O2   TYPE=O          CHARGE=-0.65480 END  !
ATOM   C3'   TYPE=CT         CHARGE= 0.31340 END  !
ATOM   C2'   TYPE=CT         CHARGE=-0.12520 END  !
ATOM  H2'1   TYPE=HC         CHARGE= 0.06110 END  !
ATOM  H2'2   TYPE=HC         CHARGE= 0.06110 END  !
ATOM   C7'   TYPE=CT         CHARGE=-0.13230 END  !
ATOM  H7'1   TYPE=HC         CHARGE= 0.04620 END  !
ATOM  H7'2   TYPE=HC         CHARGE= 0.04620 END  !
ATOM   C6'   TYPE=C6         CHARGE=-0.04910 END  !
ATOM   H6'   TYPE=HC         CHARGE= 0.11380 END  !
ATOM   C8'   TYPE=C8         CHARGE=-0.35080 END  !
ATOM  H8'1   TYPE=H8         CHARGE= 0.13530 END  !
ATOM  H8'2   TYPE=H8         CHARGE= 0.13530 END  !
ATOM   O3'   TYPE=OS         CHARGE=-0.57750 END  !
BOND    P   O1P
BOND    P   O2P
BOND    P   O5'
BOND   O5'  C5'
BOND   C5'  C4'
BOND   C5'  C6'
BOND   C5'  C8'
BOND   C4'  H4'
BOND   C4'  O4'
BOND   C4'  C3'
BOND   O4'  C1'
BOND   C1'  H1'
BOND   C1'  N1
BOND   C1'  C2'
BOND   N1   C6
BOND   N1   C2
BOND   C6   H6
BOND   C6   C5
BOND   C5   H5
BOND   C5   C4
```

```
BOND   C4   N4
BOND   C4   N3
BOND   N4  H41
BOND   N4  H42
BOND   N3   C2
BOND   C2   O2
BOND  C3'  C2'
BOND  C3'  O3'
BOND  C3'  C7'
BOND  C2'  H2'1
BOND  C2'  H2'2
BOND  C7'  H7'1
BOND  C7'  H7'2
BOND  C7'  C6'
BOND  C6'  H6'
BOND  C6'  C8'
BOND  C8'  H8'1
BOND  C8'  H8'2
IMPR   C2   C6   N1  C1'
IMPR   N1   N3   C2   O2
IMPR   C4  H41   N4  H42
IMPR   N1   C5   C6   H6
IMPR   C6   C4   C5   H5
IMPR   C5   N4   C4   N3
!Other
IMPRoper C5  C4  N4  H41
IMPRoper C5  C6  N1  C2
IMPRoper N3  C4  C5  C6
IMPRoper C2  N3  C4  C5
END

RESI TM ! Tc-MetCYT
ATOM    P   TYPE=P       CHARGE=  1.21660 END  !
ATOM  O1P   TYPE=O2      CHARGE= -0.79140 END  !
ATOM  O2P   TYPE=O2      CHARGE= -0.79140 END  !
ATOM  O5'   TYPE=OS      CHARGE= -0.49280 END  !
ATOM  C5'   TYPE=C5      CHARGE=  0.20640 END  !
ATOM  C4'   TYPE=CT      CHARGE=  0.19590 END  !
ATOM  H4'   TYPE=H1      CHARGE=  0.09370 END  !
ATOM  O4'   TYPE=OS      CHARGE= -0.45860 END  !
ATOM  C1'   TYPE=CT      CHARGE=  0.12510 END  !
ATOM  H1'   TYPE=H2      CHARGE=  0.12920 END  !
ATOM   N1   TYPE=N*      CHARGE= -0.04560 END  !
ATOM   C6   TYPE=CM      CHARGE= -0.11830 END  !
ATOM   H6   TYPE=H4      CHARGE=  0.19310 END  !
ATOM   C5   TYPE=CM      CHARGE= -0.10920 END  !
ATOM   C7   TYPE=CT      CHARGE= -0.20040 END  !
ATOM  H71   TYPE=HC      CHARGE=  0.06980 END  !
ATOM  H72   TYPE=HC      CHARGE=  0.06980 END  !
ATOM  H73   TYPE=HC      CHARGE=  0.06980 END  !
ATOM   C4   TYPE=CA      CHARGE=  0.61570 END  !
```

```
ATOM   N4    TYPE=N2      CHARGE= -0.85710 END  !
ATOM   H41   TYPE=H       CHARGE=  0.39440 END  !
ATOM   H42   TYPE=H       CHARGE=  0.39440 END  !
ATOM   N3    TYPE=NC      CHARGE= -0.72040 END  !
ATOM   C2    TYPE=C       CHARGE=  0.78250 END  !
ATOM   O2    TYPE=O       CHARGE= -0.65370 END  !
ATOM   C3'   TYPE=CT      CHARGE=  0.27860 END  !
ATOM   C2'   TYPE=CT      CHARGE= -0.07600 END  !
ATOM  H2'1   TYPE=HC      CHARGE=  0.05590 END  !
ATOM  H2'2   TYPE=HC      CHARGE=  0.05590 END  !
ATOM   C7'   TYPE=CT      CHARGE= -0.10070 END  !
ATOM  H7'1   TYPE=HC      CHARGE=  0.04060 END  !
ATOM  H7'2   TYPE=HC      CHARGE=  0.04060 END  !
ATOM   C6'   TYPE=C6      CHARGE= -0.06740 END  !
ATOM   H6'   TYPE=HC      CHARGE=  0.12560 END  !
ATOM   C8'   TYPE=C8      CHARGE= -0.40430 END  !
ATOM  H8'1   TYPE=H8      CHARGE=  0.15500 END  !
ATOM  H8'2   TYPE=H8      CHARGE=  0.15500 END  !
ATOM   O3'   TYPE=OS      CHARGE= -0.57750 END  !
BOND    P   O1P
BOND    P   O2P
BOND    P   O5'
BOND  O5'  C5'
BOND  C5'  C4'
BOND  C5'  C6'
BOND  C5'  C8'
BOND  C4'  H4'
BOND  C4'  O4'
BOND  C4'  C3'
BOND  O4'  C1'
BOND  C1'  H1'
BOND  C1'   N1
BOND  C1'  C2'
BOND   N1   C6
BOND   N1   C2
BOND   C6   H6
BOND   C6   C5
BOND   C5   C7
BOND   C7   H71
BOND   C7   H72
BOND   C7   H73
BOND   C5   C4
BOND   C4   N4
BOND   C4   N3
BOND   N4  H41
BOND   N4  H42
BOND   N3   C2
BOND   C2   O2
BOND  C3'  C2'
BOND  C3'  O3'
BOND  C3'  C7'
```

```
BOND   C2'  H2'1
BOND   C2'  H2'2
BOND   C7'  H7'1
BOND   C7'  H7'2
BOND   C7'  C6'
BOND   C6'  H6'
BOND   C6'  C8'
BOND   C8'  H8'1
BOND   C8'  H8'2
IMPR    C2    C6    N1   C1'
IMPR    N1    N3    C2    O2
IMPR    C4   H41    N4   H42
IMPR    N1    C5    C6    H6
IMPR    C4    C6    C5    C7
IMPR    C5    N4    C4    N3
IMPR    N1    C6    C5    C7
!Other
IMPRoper C5   C4   N4   H41
IMPRoper N3   C4   N4   H42
IMPRoper C5   C6   N1   C2
IMPRoper N3   C4   C5   C6
IMPRoper C2   N3   C4   C5
END

RESI TM5 ! Tc-MetCYT 5'terminal
ATOM   H5T    TYPE=HO         CHARGE=  0.42470 END  !
ATOM   O5'    TYPE=OH         CHARGE= -0.60250 END  !
ATOM   C5'    TYPE=C5         CHARGE=  0.20640 END  !
ATOM   C4'    TYPE=CT         CHARGE=  0.19590 END  !
ATOM   H4'    TYPE=H1         CHARGE=  0.09370 END  !
ATOM   O4'    TYPE=OS         CHARGE= -0.45860 END  !
ATOM   C1'    TYPE=CT         CHARGE=  0.12510 END  !
ATOM   H1'    TYPE=H2         CHARGE=  0.12920 END  !
ATOM    N1    TYPE=N*         CHARGE= -0.04560 END  !
ATOM    C6    TYPE=CM         CHARGE= -0.11830 END  !
ATOM    H6    TYPE=H4         CHARGE=  0.19310 END  !
ATOM    C5    TYPE=CM         CHARGE= -0.10920 END  !
ATOM    C7    TYPE=CT         CHARGE= -0.20040 END  !
ATOM   H71    TYPE=HC         CHARGE=  0.06980 END  !
ATOM   H72    TYPE=HC         CHARGE=  0.06980 END  !
ATOM   H73    TYPE=HC         CHARGE=  0.06980 END  !
ATOM    C4    TYPE=CA         CHARGE=  0.61570 END  !
ATOM    N4    TYPE=N2         CHARGE= -0.85710 END  !
ATOM   H41    TYPE=H          CHARGE=  0.39440 END  !
ATOM   H42    TYPE=H          CHARGE=  0.39440 END  !
ATOM    N3    TYPE=NC         CHARGE= -0.72040 END  !
ATOM    C2    TYPE=C          CHARGE=  0.78250 END  !
ATOM    O2    TYPE=O          CHARGE= -0.65370 END  !
ATOM   C3'    TYPE=CT         CHARGE=  0.27860 END  !
ATOM   C2'    TYPE=CT         CHARGE= -0.07600 END  !
ATOM  H2'1    TYPE=HC         CHARGE=  0.05590 END  !
```

```
ATOM  H2'2    TYPE=HC          CHARGE=  0.05590 END  !
ATOM   C7'    TYPE=CT          CHARGE= -0.10070 END  !
ATOM  H7'1    TYPE=HC          CHARGE=  0.04060 END  !
ATOM  H7'2    TYPE=HC          CHARGE=  0.04060 END  !
ATOM   C6'    TYPE=C6          CHARGE= -0.06740 END  !
ATOM   H6'    TYPE=HC          CHARGE=  0.12560 END  !
ATOM   C8'    TYPE=C8          CHARGE= -0.40430 END  !
ATOM  H8'1    TYPE=H8          CHARGE=  0.15500 END  !
ATOM  H8'2    TYPE=H8          CHARGE=  0.15500 END  !
ATOM   O3'    TYPE=OS          CHARGE= -0.57750 END  !
BOND   H5T   O5'
BOND   O5'   C5'
BOND   C5'   C4'
BOND   C5'   C6'
BOND   C5'   C8'
BOND   C4'   H4'
BOND   C4'   O4'
BOND   C4'   C3'
BOND   O4'   C1'
BOND   C1'   H1'
BOND   C1'    N1
BOND   C1'   C2'
BOND    N1    C6
BOND    N1    C2
BOND    C6    H6
BOND    C6    C5
BOND    C5    C7
BOND    C7    H71
BOND    C7    H72
BOND    C7    H73
BOND    C5    C4
BOND    C4    N4
BOND    C4    N3
BOND    N4   H41
BOND    N4   H42
BOND    N3    C2
BOND    C2    O2
BOND   C3'   C2'
BOND   C3'   O3'
BOND   C3'   C7'
BOND   C2'  H2'1
BOND   C2'  H2'2
BOND   C7'  H7'1
BOND   C7'  H7'2
BOND   C7'  C6'
BOND   C6'  H6'
BOND   C6'  C8'
BOND   C8'  H8'1
BOND   C8'  H8'2
IMPR    C2    C6    N1   C1'
IMPR    N1    N3    C2    O2
```

```
IMPR    C4   H41    N4   H42
IMPR    N1    C5    C6    H6
IMPR    C4    C6    C5    C7
IMPR    C5    N4    C4    N3
IMPR    N1    C6    C5    C7
!Other
IMPRoper C5   C4   N4   H41
IMPRoper N3   C4   N4   H42
IMPRoper C5   C6   N1   C2
IMPRoper N3   C4   C5   C6
IMPRoper C2   N3   C4   C5
END


RESI TM3 ! Tc-MetCYT 3' terminal
ATOM     P    TYPE=P          CHARGE=  1.21660 END  !
ATOM   O1P    TYPE=O2         CHARGE= -0.79140 END  !
ATOM   O2P    TYPE=O2         CHARGE= -0.79140 END  !
ATOM   O5'    TYPE=OS         CHARGE= -0.49280 END  !
ATOM   C5'    TYPE=C5         CHARGE=  0.20640 END  !
ATOM   C4'    TYPE=CT         CHARGE=  0.19590 END  !
ATOM   H4'    TYPE=H1         CHARGE=  0.09370 END  !
ATOM   O4'    TYPE=OS         CHARGE= -0.45860 END  !
ATOM   C1'    TYPE=CT         CHARGE=  0.12510 END  !
ATOM   H1'    TYPE=H2         CHARGE=  0.12920 END  !
ATOM    N1    TYPE=N*         CHARGE= -0.04560 END  !
ATOM    C6    TYPE=CM         CHARGE= -0.11830 END  !
ATOM    H6    TYPE=H4         CHARGE=  0.19310 END  !
ATOM    C5    TYPE=CM         CHARGE= -0.10920 END  !
ATOM    C7    TYPE=CT         CHARGE= -0.20040 END  !
ATOM   H71    TYPE=HC         CHARGE=  0.06980 END  !
ATOM   H72    TYPE=HC         CHARGE=  0.06980 END  !
ATOM   H73    TYPE=HC         CHARGE=  0.06980 END  !
ATOM    C4    TYPE=CA         CHARGE=  0.61570 END  !
ATOM    N4    TYPE=N2         CHARGE= -0.85710 END  !
ATOM   H41    TYPE=H          CHARGE=  0.39440 END  !
ATOM   H42    TYPE=H          CHARGE=  0.39440 END  !
ATOM    N3    TYPE=NC         CHARGE= -0.72040 END  !
ATOM    C2    TYPE=C          CHARGE=  0.78250 END  !
ATOM    O2    TYPE=O          CHARGE= -0.65370 END  !
ATOM   C3'    TYPE=CT         CHARGE=  0.27860 END  !
ATOM   C2'    TYPE=CT         CHARGE= -0.07600 END  !
ATOM  H2'1    TYPE=HC         CHARGE=  0.05590 END  !
ATOM  H2'2    TYPE=HC         CHARGE=  0.05590 END  !
ATOM   C7'    TYPE=CT         CHARGE= -0.10070 END  !
ATOM  H7'1    TYPE=HC         CHARGE=  0.04060 END  !
ATOM  H7'2    TYPE=HC         CHARGE=  0.04060 END  !
ATOM   C6'    TYPE=C6         CHARGE= -0.06740 END  !
ATOM   H6'    TYPE=HC         CHARGE=  0.12560 END  !
ATOM   C8'    TYPE=C8         CHARGE= -0.40430 END  !
```

```
ATOM  H8'1    TYPE=H8          CHARGE=  0.15500 END  !
ATOM  H8'2    TYPE=H8          CHARGE=  0.15500 END  !
ATOM   O3'    TYPE=OH          CHARGE= -0.57750 END  !
ATOM  H3T     TYPE=HO          CHARGE=  0.44190 END  !
BOND     P   O1P
BOND     P   O2P
BOND     P   O5'
BOND   O5'   C5'
BOND   C5'   C4'
BOND   C5'   C6'
BOND   C5'   C8'
BOND   C4'   H4'
BOND   C4'   O4'
BOND   C4'   C3'
BOND   O4'   C1'
BOND   C1'   H1'
BOND   C1'    N1
BOND   C1'   C2'
BOND    N1    C6
BOND    N1    C2
BOND    C6    H6
BOND    C6    C5
BOND    C5    C7
BOND    C7    H71
BOND    C7    H72
BOND    C7    H73
BOND    C5    C4
BOND    C4    N4
BOND    C4    N3
BOND    N4   H41
BOND    N4   H42
BOND    N3    C2
BOND    C2    O2
BOND   C3'   C2'
BOND   C3'   O3'
BOND   C3'   C7'
BOND   C2'  H2'1
BOND   C2'  H2'2
BOND   C7'  H7'1
BOND   C7'  H7'2
BOND   C7'  C6'
BOND   C6'  H6'
BOND   C6'  C8'
BOND   C8'  H8'1
BOND   C8'  H8'2
BOND   O3'  H3T
IMPR    C2    C6    N1   C1'
IMPR    N1    N3    C2    O2
IMPR    C4   H41    N4   H42
IMPR    N1    C5    C6    H6
IMPR    C4    C6    C5    C7
```

```
IMPR    C5    N4    C4    N3
IMPR    N1    C6    C5    C7
!Other
IMPRoper C5   C4   N4   H41
IMPRoper N3   C4   N4   H42
IMPRoper C5   C6   N1   C2
IMPRoper N3   C4   C5   C6
IMPRoper C2   N3   C4   C5
END

BOND C6   CT   310.0 1.516 ! Kr same as CT-CT, bond length QM
BOND C6   C8   310.0 1.497 ! Kr same as CT-CT, bond length QM
BOND C6   C5   310.0 1.497 ! Kr same as CT-CT, bond length QM
BOND C5   C8   310.0 1.497 ! Kr same as CT-CT, bond length QM
BOND C5   OS   320.0 1.410 ! Kr same as CT-OS, bond length QM
BOND C5   OH   320.0 1.410 ! Kr same as CT-OH, bond length QM
BOND C5   CT   310.0 1.516 ! Kr same as CT-CT, bond length QM
BOND CT   C8   310.0 1.526 ! Kr same as CT-CT, bond length QM
BOND C8   H8   340.0 1.090 ! same as CT-HC
BOND C6   HC   340.0 1.090 ! same as CT-HC
BOND C5   HC   340.0 1.090 ! same as CT-HC

ANGL C8   C5    C6    160.0 60.00 !  K0 same as CT-CT-CT, angle QM
ANGL C5   C8    C6    160.0 60.00 ! K0 same as CT-CT-CT, angle QM
ANGL C8   C6    C5    160.0 60.00 ! K0 same as CT-CT-CT, angle QM
ANGL C6   C5    CT    150.0 109.50 ! K0 same as CT-CT-CT, angle QM
ANGL C6   CT    CT    150.0 109.50 ! K0 same as CT-CT-CT, angle QM
ANGL C5   CT    CT    150.0 109.50 ! K0 same as CT-CT-CT, angle QM
ANGL C5   C6    CT    150.0 109.50 ! K0 same as CT-CT-CT, angle QM
ANGL C6   C5    OS    150.0 119.50 ! K0 same as CT-CT-OS, angle QM
ANGL C8   C5    OS    150.0 119.50 ! K0 same as CT-CT-OS, angle QM
ANGL C5   CT    OS    150.0 109.50 ! K0 same as CT-CT-OS angle QM
ANGL OS   C5    CT    150.0 119.50 ! K0 same as CT-CT-OS, angle QM
ANGL C8   C5    CT    150.0 116.50 ! K0 same as CT-CT-CT, angle QM
ANGL C8   C6    CT    150.0 116.50 ! K0 same as CT-CT-CT, angle QM
ANGL H8   C8    H8    150.0 115.20 ! K0 same as HC-CT-HC, angle QM
ANGL C5   C8    H8    160.0 115.80 ! K0 same as CT-CT-HC, angle QM
ANGL C6   C8    H8    160.0 117.70 ! K0 same as CT-CT-HC, angle QM
ANGL C5   C6    HC    160.0 119.50 ! K0 same as CT-CT-HC, angle QM
ANGL C8   C6    HC    160.0 119.50 ! K0 same as CT-CT-HC, angle QM
ANGL CT   C6    HC    160.0 119.70 ! K0 same as CT-CT-HC, angle QM
ANGL HC   CT    C6    160.0 109.50 ! K0 same as CT-CT-HC, angle QM
```


1. Frisch, M.J., Trucks, G.W., Schlegel, H.B., Scuseria, G.E., Robb, M.A., Cheeseman, J.R., Scalmani, G., Barone, V., Petersson, G.A., Nakatsuji, H. *et al.* (2009) Gaussian 16. *Gaussian, Inc., Wallingford CT*.
2. Dupradeau, F.Y., Pigache, A., Zaffran, T., Savineau, C., Lelong, R., Grivel, N., Lelong, D., Rosanski, W. and Cieplak, P. (2010) The R.E.D. tools: advances in RESP and ESP charge derivation and force field library building. *Physical chemistry chemical physics : PCCP*, **12**, 7821-7839.
3. Cieplak, P., Cornell, W.D., Bayly, C. and Kollman, P.A. (1995) Application of the multimolecule and multiconformational RESP methodology to biopolymers: Charge derivation for DNA, RNA, and proteins. *Journal of Computational Chemistry*, **16**, 1357-1377.
